# *Withania somnifera*: Progress towards a Pharmaceutical Agent for Immunomodulation and Cancer Therapeutics

**DOI:** 10.3390/pharmaceutics14030611

**Published:** 2022-03-10

**Authors:** Vivek K. Kashyap, Godwin Peasah-Darkwah, Anupam Dhasmana, Meena Jaggi, Murali M. Yallapu, Subhash C. Chauhan

**Affiliations:** 1 Department of Immunology and Microbiology, School of Medicine, University of Texas Rio Grande Valley, McAllen, TX 78504, USA; vivek.kashyap@utrgv.edu (V.K.K.); godwin.peasahdarkwah01@utrgv.edu (G.P.-D.); anupam.dhasmana@utrgv.edu (A.D.); meena.jaggi@utrgv.edu (M.J.); 2 South Texas Center of Excellence in Cancer Research, School of Medicine, University of Texas Rio Grande Valley, McAllen, TX 78504, USA

**Keywords:** ashwagandha, *Withania somnifera*, Withanolides, Withanolide D, nanoformulation

## Abstract

Chemotherapy is one of the prime treatment options for cancer. However, the key issues with traditional chemotherapy are recurrence of cancer, development of resistance to chemotherapeutic agents, affordability, late-stage detection, serious health consequences, and inaccessibility. Hence, there is an urgent need to find innovative and cost-effective therapies that can target multiple gene products with minimal adverse reactions. Natural phytochemicals originating from plants constitute a significant proportion of the possible therapeutic agents. In this article, we reviewed the advances and the potential of *Withania somnifera* (WS) as an anticancer and immunomodulatory molecule. Several preclinical studies have shown the potential of WS to prevent or slow the progression of cancer originating from various organs such as the liver, cervix, breast, brain, colon, skin, lung, and prostate. WS extracts act via various pathways and provide optimum effectiveness against drug resistance in cancer. However, stability, bioavailability, and target specificity are major obstacles in combination therapy and have limited their application. The novel nanotechnology approaches enable solubility, stability, absorption, protection from premature degradation in the body, and increased circulation time and invariably results in a high differential uptake efficiency in the phytochemical’s target cells. The present review primarily emphasizes the insights of WS source, chemistry, and the molecular pathways involved in tumor regression, as well as developments achieved in the delivery of WS for cancer therapy using nanotechnology. This review substantiates WS as a potential immunomodulatory, anticancer, and chemopreventive agent and highlights its potential use in cancer treatment.

## 1. Introduction

Cancer is only second to heart disease as the leading cause of mortality globally. In 2022, the American Cancer Society reported about 1,918,030 new cases of cancer and 609,360 cancer-related mortalities in the United States alone [1]. Over the past three decades, significant progress in the detection and treatment of cancers has altered early diagnosis, prevention, and therapeutic strategies resulting in a decline in the mortality rate. The cancer mortality rate reduced by 27% between 2001 and 2020. Male cancer death rates decreased by 30% and female death rates decreased by 25%, although male cancer death rates remained higher (170.3 deaths per 100,000 people) than female death rates (124.5 deaths per 100,000 people) [2]. Chemotherapy, radiation therapy, surgery, immunotherapy, and targeted therapy are being used to treat cancer. However, many chemotherapeutic methods are linked with severe side effects and resistance within a few months of therapy. Commercially available Food and Drug Administration (FDA)-approved drugs mostly target a single gene or pathway [3,4,5,6]. As a result, there is a growing need to find new anticancer molecules/medicines that could target multiple targets in cancer while having minimal adverse effects. Recently, many plant-based agents such as alkaloids, have demonstrated pronounced anti-cancer activity, either as a single agent or as a combination regimen with standard anti-cancer agents [7]. The majority of characterized phytochemicals have exhibited chemo- and radio-sensitizing activity in chemo and radio-resistant tumor cells [7]. About 40% of the FDA-approved drugs in the market are based on plant products, 74% of which are anticancer drugs [7,8,9]. Moreover, the majority of these phytochemicals actively target tumor cells specifically and pose minimal toxicity to healthy cells. 

*Withania somnifera* (WS) is a woody, evergreen shrub, roughly 0.5 to 2.0 m tall, and in English it is called “Winter cherry” or “Indian Ginseng”, in Sanskrit “Ashwagandha”, in Hindi “Asgandh”, and in Urdu “Asgand” [10,11]. The name “Ashwagandha” originates from the plant’s roots, which have the distinctive smell of a wet horse (“ashwa” means horse and “gandha” means smell). For more than 5000 years, the complete plant or various portions of WS have been employed in India’s Ayurvedic and Unani medical systems for medicinal and therapeutic purposes, and the plant was reported as an official drug in the Indian Pharmacopoeia-1985 [10]. 

WS is cytotoxic to a variety of tumor cells but has minimal effect on healthy human cells, indicating its specific effects on cancer cells [12]. WS has been demonstrated to have anxiolytic, antiangiogenic, antidepressive, anti-metastatic, anti-tumoral, cytotoxic, genotoxic, antibacterial, antifungal, and antidiabetic properties in several in vitro and in vivo experiments [13,14,15]. WS modulates cytotoxicity in cancer cells by accumulating intracellular reactive oxygen species (ROS) [16,17,18]. WS inhibits several aberrant pathways implicated in inflammation and proliferation (e.g., IL-6, TNF-α, and cycloxygenase-2 (COX-2)), angiogenesis and metastasis (e.g., VEGF, MMP9, TWIST, NF-κB, and STAT), cell survival (e.g., Bcl-2, Bcl-xL, survivin, and cIAP1/2), and regulation of the cell cycle (e.g., cyclin A, cyclin D1, Cdks, p21, and p53) [12,13,14,19,20,21,22,23,24,25,26,27,28]. Furthermore, WS is an adaptogenic Ayurvedic plant, which is often used to counteract and relieve stress, ultimately improving overall well-being, and numerous studies have shown the use of WS for stress resistance [29,30,31]. A high-concentration full-spectrum WS root extract enhances an individual’s resistance to stress and, as a result, their self-assessed quality of life [31,32]. In this review, we discuss the chemical properties, pharmacokinetics, anticancer potential, toxicity, and pharmacological significance of WS. We also highlight the molecular pathways of WS contributing towards anticancer activity, combinatorial therapy, and the chemo/radio-sensitizing effects. The phytochemical properties of WS would yield novel insights and establish the basics for clinical investigations to develop WS as an anti-cancer medication.

## 2. Biological and Chemical Properties of *Withania somnifera*

### 2.1. Sources and Chemical Properties of Withania somnifera

The 23 known Withania species are widely distributed in the arid regions of India, Baluchistan, Afghanistan, Sri Lanka, Congo, South Africa, Egypt, Morocco, and Jordan [33]. WS plant organs such as the root, leaf, fruit, and seed possess adequate bio-active chemicals that have been implicated in pronounced anticancer, anti-microbial, cardioprotective, and neuroprotective mechanisms [33,34,35,36,37,38,39,40,41,42].

**Figure 1:**
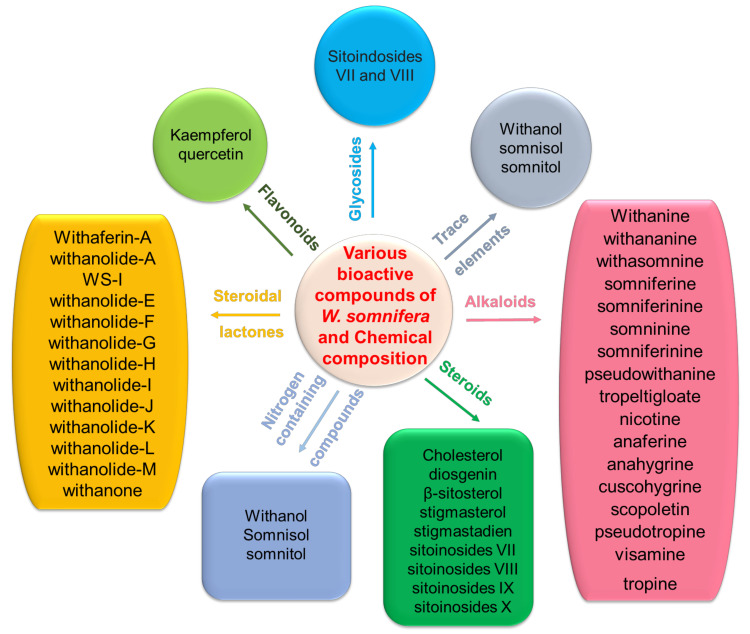
Various phytochemicals present in *Withania somnifera*.

The presence of withanolides, a group of steroidal lactones, is thought to be responsible for the pharmacological activity of WS roots [43]. WS has demonstrated non-medicinal properties, such as enhanced memory and cognition, mood elevation, and rejuvenation [29]. WS also serves as an energy-boosting tonic called Medharasayana, meaning “enhanced memory and learning.” Laboratory studies have shown that the roots of WS contain about 35 phytoconstituents [44]. The main physiologically active chemical molecules present are alkaloids (isopellertierine and anferine), steroidal lactones (withanolides, withaferins saponins with additional acyl group (sitoindoside VII and VIII), and withanoloides with C-27 linked to glucose (sitonidoside XI and X). A chemo assessment of Ashwagandha revealed that its primary ingredients are alkaloids and steroidal lactones. Withanine is the most abundant out of all the numerous alkaloids. Others include somniferine, pseudo-withanine, tropine, pseudo-tropine, 3-a-gloyloxytropine, choline, anaferine, anahydrine, etc. Withanolides present in leaf possess a C28 steroidal nucleus, a C9 side chain, and a hexagonal lactone ring. WS has been investigated for its twelve alkaloids, 35 withanolides, and seven sitoindosides. The majority of Ashwagandha’s pharmacological action has been ascribed to two major withanolides, Withaferin A and Withanolide D [45,46]. The fresh plant of WS is rich in fatty acids, fatty alcohols, volatile oils, and hydrocarbons, including myristic acid, palmitate, linoleic acid, and the straight chain hydrocarbon hexatriacontane [47]. The roots of ashwagandha include alkaloids (wide variation of 0.13–0.31%), starch, reducing carbohydrates, hentriacontane, glycosides, dulcitol, withaniol acid, and a neutral molecule. In addition, the leaves contain free amino acids including aspartic acid, glycine, tyrosine, alanine, proline, tryptophan, glutamic acid, and cystine. Fruits of Ashwagandha harbor a milk-coagulating enzyme, two esterases, free amino acids, fatty oil, essential oils, and alkaloids. The bioactive chemicals extracted from WS is listed in Figure 1 and their anticancer functions are presented in Table 1.

### 2.2. Toxicity of Withania somnifera

Due to low cost and abundance, medicinal plants have been of considerable pharmacological interest, particularly in cancer prevention. Various studies have shown that natural bioactive drugs with minimal toxicity possess immense therapeutic potential. However, safety issues have often been highlighted and must be addressed before they are utilized as immunomodulatory and anticancer agents. Traditional chemotherapies pose several side effects, including those that impair the functioning of numerous organs, such as the heart, liver, and kidneys. Research reports have emphasized the preventative activity of Withaferin A against bromobenzene-induced liver and kidney breakdown in mice [48]. This was evident from the reduced levels of liver and kidney biomarkers, lipid peroxidation, and cytokines (TNF-α and IL-1), after a 10 mg/kg pre-treatment dose of Withaferin A administered in mice. In the Withaferin A pre-treatment mice group, there were low levels of cytokines with reduced oxidative stress; mitochondrial impairment was prevented and the equilibrium between Bax/Bcl-2 was restored [48]. 

According to a recent study, Withaferin A increased the capacity of H9c2 cells to survive against simulated ischemia/reperfusion (SI/R) or hydrogen peroxide (H_2_O_2_)-induced cell death in myocardial ischemia reperfusion (MI/R) damage, as well as inhibiting the increased oxidative stress caused by SI/R [49]. In addition, Withaferin A effectively inhibited H_2_O_2_-induced overexpression of SOD2, SOD3, and Prdx-1, which improved cardiomyocyte caspase-3 activity in an Akt-dependent way [49]. Furthermore, Withaferin A reduced cerulein-induced acute pancreatitis due to oxidative stress and inflammation [50]. Increased tissue malondialdehyde (MDA), NO, and myeloperoxidase and nitrotyrosine expression in the parameters assessed contribute to the notion that oxidative stress and inflammation play a role in acute pancreatitis [50]. Furthermore, Withaferin A also decreased the acetaminophen-mediated liver damage in mice in an Nrf2-dependent manner, typical of a stress-responsive transcription factor and a well-established chemoprevention target. Withaferin A increased nuclear factor-erythroid factor 2-related factor 2 (Nrf2) signaling in a non-canonical Keap-independent, Pten/PI3k/Akt-dependent way in this research [51]. Studies indicate that Withaferin A performs an antifibrotic action in scleroderma by inhibiting pro-inflammatory fibrosis involving Transforming growth factor-b1(TGF-β)/Smad signaling and substantially reducing fibroblast conversion to myofibroblasts. Also, the FoxO3a-Akt-dependent nuclear factor-kappa B (NF-κB)/IKK-regulated inflammatory cascade, which is a key signaling mechanism in fibrogenesis, is modulated by Withaferin A [52]. In a recent research study, tumor-targeting silver nanoparticles (Ag NP) were used to produce NP-related macrophage toxicity. When nanoparticles (NPs) were given in combination with WS root extract (35 mg/kg), the toxic effects in rats were significantly reduced [53]. Despite these fundamental and mechanistic investigations, the potential of WS extracts as dietary supplements has only been explored in one study, which included 100 breast cancer patients undergoing chemotherapies (used as complementary). It was shown that the tiredness associated with treatment reduced and the overall quality of life improved after supplementation with WS extracts [54].

**Table 1:** Different bioactive molecules of *Withania somnifera* and their anticancer activities.

Bioactive Molecules	Part Used	Type of Cancer	Medicinal Value of the Bioactive Molecules	Ref.
Withaferin A	Leaves	Breast cancer cell lines MDA-MB-231 and MCF-7	In vitro, WA inhibited the expression of ER, RET, and HSF1 while increasing the expression of phospho-p38 MAPK, p53, and p21 in MCF-7 breast cancer cells	[19]
		Breast cancer cells and mice model	Inhibit cell proliferation, reduced tumor growth and induce FOXO3a- and Bim-dependent apoptosis	[20]
		Ovarian cancer cells	Inhibits cell growth, inducing apoptosis, and cell cycle arrest and targeting Notch1 and Notch3 down regulates	[55]
		Breast cancer cell lines, 4T1 (mouse breast), Nu/nu mice, Balb/c mice, SCID mice	Chemoprevention and reduced tumor growth	[56]
		Leukemia U937 cells	Induces apoptosis by activating caspase 3, JNK, and Akt signaling pathways	[57]
		Human renal cancer cells (Caki cells)	Increased radiation-induced apoptosis by ROS generation, inhibits the expression of Bcl-2 and dephosphorylation of Akt	[21]
		Human cancer cell lines Balb/c nude mice	Increased expression of p53 transcription factor, suppression of tumour growth and apoptosis	[58]
		Osteogenic sarcoma (U2OS) and fibrosarcoma (HT1080) cells	In vitro and in vivo anticancer activity	[59]
	Root	Balb/C mice	WA reduced macrophage production of pro-inflammatory cytokines, tumor weight, granulocytic MDSC number, and MDSC potential to inhibit antigen-driven activation of CD4+ and CD8+ T cells	[22]
		Prostate PC-3 xenografts in nude mice	Inhibition of the proteasomal chymotrypsin-like activity and tumor growth	[60]
		DRO81-(medullary thyroid) and nu/nu mice	Reduced tumor growth and inhibited total and phospho-RET levels at the protein level	[61]
		Malignant pleural mesothelioma (MPM), MPM (H2373, H2452, H2461, H226 and AB12) cells and BALB/c mice	Inhibits the proteasome activity in mesothelioma in vitro and in vivo and reduces tumor growth	[62]
		Pancreatic cancer cell lines Panc-1, MiaPaCa2 and BxPc3 and Panc-1 xenografts mice	Showed potent cytotoxicity against pancreatic cancer cells *in vitro*, reduced tumor growth and targeted heat shock protein 90	[63]
		7,12-dimethylbenz[a]anthracene (DMBA) induced oral carcinogenesis in Syrian golden hamsters	Exhibit anti-lipid peroxidative and antioxidant activity	[64]
		Breast cancer cell lines MCF-7 and SUM159	Exhibit antiproliferative activity and Induce apoptosis	[65]
	Root	Sarcoma 180, Animal model	Reduced tumor growth	[66]
	NR	Cervical cancer cells CaSki, HeLa, SiHa, C33a and athymic nu/nu mice	WA significantly reduced tumor growth inhibit expression of HPV E6/E7 oncogenes and restores the p53 and induces apoptosis	[67]
		Breast cancer cell lines and MMTV-*neu* mice	Inhibit the expression of aldehyde dehydrogenase (ALDH1), CD44 (high)/CD24 (low)/epithelial-specific antigen-positive (ESA+) along with Oct4, SOX-2, Nanog mRNA and inhibition of cancer stem cell growth	[23]
		Colon cancer cell lines C57BL/6-APC*^Min^^/^^+^*	Inhibit the expression of interleukin-6, COX-2, TNF-α, pAKT, Notch1, NF-κB and Ki67	[24]
		Colorectal cancer cells HCT-116 and RKO	Exhibit cell cycle arrest and ROS-dependent mitochondrial dysfunction-mediated apoptosis	[68]
	Root	Gliobastoma multiforme, nu/nu mice	GBM neurosphere collapsed at nM concentrations	[69]
	NR	Panc-1, SW1990, MIAPaCa-2, AsPC-1 and BxPc-3 and xenografts mouse model	Reduced tumor growth, activation of proteasome inhibition, and enhancement of ubiquitinated protein accumulation, resulting in ER stress-mediated apoptosis	[70]
	Leaves	Lymphoid and myeloid leukemia cells	Induces mitochondrial apoptosis by activating the p38 MAPK cascade	[71]
		Myeloid leukemia HL-60 cells	Early ROS generation and mitochondrial dysfunction	[72]
	NR	Prostate cancer cells and PC-3 xenografts	Par-4-Dependent Apoptosis	[73]
	Fruit	Liver cancer cells HepG2	Remarkable changes in the chromatin structure (fragmentation, uniform condensation)	[74]
	Root	HUVEC cells	Inhibition of NF-κB by interference with the ubiquitin-mediated proteasome pathway by increasing levels of poly-ubiquitinated proteins	[75]
	Leaves	HepG2 (hepatocellular carcinoma)	Increased the expression of Caspase-3; caspase-8, caspase-9, upregulated antioxidant activity and decreased TNF-α level	[76]
		Telomerase plus, telomerase negative, ALT (JFCF-1 l and JFCF-4D)	Exhibit cytotoxicity, cause DNA damage, and promote telomere dysfunction	[77]
	NR	Melanoma cells (Lu1205, M14, Mel501 and SK28)	Inhibit cell proliferation, induces apoptosis; downregulated ROS productions and Bcl-2 expression	[78]
		NSCLC cancer cell lines A549, CL141, H441, CL97, H1975, CL152, and H1299 and NOD/SCID mice	Reduced lung CSC growth and spheroid formation capacity, mTOR/STAT3 signaling downregulation, and EGFR inhibition	[24,79]
		B cell lymphoma cell line (Lymphatic systems) and Balb/c mice	Decreased cell survival, heat shock protein (Hsp) 90, key kinases and cell cycle regulators	[80]
		Human A549 and U937 cells	Inhibited cell adhesion and reduces the expression of ICAM-1 and VCAM-1 TNF-α and NF-κB	[25]
		Prostate cancer cell lines (PC-3; DU-145 LNCaP)	Promoted cell death and inhibited the expression of c-Fos and heat-shock proteins (HSPs)	[81]
	Leaves	Colorectal cancer cell lines (SW480 and HCT116)	Inhibited cell proliferation, induce apoptosis, cell cycle G2/M arrest and associated with proteasomal degradation of Mad2 and Cdc20	[82]
Withaferin A and withanone	NR	U2OS (osteosarcoma) and TIG (normal skin fibroblast) cells	Reduced cell viability and induces p53 expression	[83]
Withaferin A and CAPE	NR	Human ovarian cancer (SKOV3 and OKV-18 and SKGII, SKGIIIb, ME180) and cervical (HeLa) cancer cells	Exhibited antiproliferative activity and induced apoptosis, increased p53, and downregulated mortalin	[84]
Withaferin A and withanone	NR	Hepatocarcinoma HUH-6 and HUH-7 cells	Reduced cell viability and induces apoptosis	[85]
27-acetoxy-4b,6a-dihydroxy-5b-chloro-1-oxowitha-2, 24-dienolide. 5b,6b,14a,15a-diepoxy 4b,27-dihydroxy-1- oxowitha-2,24-dienolide & Withaferin A	Fresh aerial parts	Lung cancer cell line NCI-H460	Anti-cancer efficacy against human lung cancer cells and growth inhibition	[86]
L-asparaginase	Fruits	Human leukemia cells	Exhibited inhibitory effect against lymphoblastic leukemia	[87]
Withaferin A and Withanolide D	Root	B16F-10 melanoma cells in C57BL/6 mice	Exhibited significant antitumor activity	[88]
Withanolide A	Root	Balb/C mice	Upregulated the Th1 response, CD4 and CD8 numbers, and enhances the activity of natural killer (NK) cells	[89]
Withanolide A, Withanoside IV, and Withanoside VI	Root	Human neuroblastoma SH-SY5Y cell line	Activate neurite outgrowth in the SH-SY5Y cell line	[90]
Triethylene glycol	Leaves	Different human cancer cells and CD1-ICR mice and Balb/c nude mice	ASH-WEX and TEG are selectively cytotoxic to cancer cells and activate the tumor suppressor proteins p53 and pRB	[91]
27-desoxy-24,25-dihydrowithaferin A	Leaves	Lung (NCI-H460), colon (HCT-116), central nervous system (SF-268) and breast (MCF-7) human tumor cell lines	Reduced viability and inhibited cell proliferation	[92]
27-Oglucopyranosylviscosalactone B	Leaves	Lung (NCI-H460), colon (HCT-116), central nervous system (SF-268) and breast (MCF-7) human tumor cell lines	Reduced viability and inhibited cell proliferation	[92]
3-azido withaferin A	NR	Human cervical (HeLa and prostate (PC-3) cancer cells and C57/BL6J mice	By modulating extracellular Par-4, it prevents cancer cell invasion and angiogenesis	[93]
4,16-dihydroxy-5h,6h-epoxyphysagulin D	Leaves	Lung (NCI-H460), colon (HCT-116), central nervous system (SF-268) and breast (MCF-7) human tumor cell lines	Reduced viability and inhibited cell proliferation	[92]
4β-Hydroxywithanolide E	Aerial parts (stems and leaves)	Human breast cancer cells (MDA-MB-231 and MCF-7)	Inhibition of NF-κB activation	[94]
4β-hydroxywithanolide E, Withaferin A	NR	Triple-negative breast cancer (TNBC) MDA-MB-231 cells	Inhibit cell viability, cell cycle arrest and apoptosis/necrosis	[95]
Combination of cucurbitacin B and withanone CucWi-N	NR	A549; TIG-3 and athymic balb/c nude mice	Reduced tumor growth, induces cellular senescence and decreases the expression of Cyclin E, Lamin A/C, CDK2, Cyclin D, CDK4, phosphorylated RB, mortalin and an increase in p53	[26]
Diacetylwithaferin A	Leaves	Lung (NCI-H460), colon (HCT-116), central nervous system (SF-268) and breast (MCF-7) human tumor cell lines	Inhibition of cell proliferation and decrease the expression of COX-2	[92]
Physagulin D (1→ 6)-h-Dglucopyranosyl-(1→4)-h-Dglucopyranoside	Leaves	Lung (NCI-H460), colon (HCT-116), central nervous system (SF-268) and breast (MCF-7) human tumor cell lines	Inhibition of cell proliferation reduces viability and decrease the expression of COX-2	[92]
Viscosalactone B	Leaves	Lung (NCI-H460), colon (HCT-116), central nervous system (SF-268) and breast (MCF-7) human tumor cell lines	Inhibition of cell proliferation reduces viability and decreases the expression of COX-2	[92]
27-desoxy-24,25-dihydrowithaferin A	Leaves	Lung (NCI-H460), colon (HCT-116), central nervous system (SF-268) and breast (MCF-7) tumor cell lines	Inhibition of cell proliferation and reduced viability	[92]
Withanolide analogue	NR	Breast cancer cells (SK-Br-3 and MCF7/BUS)	Inhibition of cell proliferation and upregulation the expression of β-tubulin	[96]
Withanolide D	Leaves	Myeloid (K562) and lymphoid (MOLT-4) cells	Induced apoptosis and cell killing through JNK and p38MAPK activation	[97]
Withanolide D	Root	Multiple myeloma CSCs and RPMI 8226 cell	Inhibition of cell proliferation and cell death induces apoptosis	[98]
Withanone and withaferin A (20:1)	NR	Metastatic cancer cells A172, IMR32, YKG1, MCF7, HT1080, U20S and Nude mice	Inhibition of cell proliferation and downregulation the expression of hnRNP-K, VEGF, and metalloproteases	
Withanoside IV	Leaves	Lung (NCI-H460), colon (HCT-116), central nervous system (SF-268) and breast (MCF-7) human tumor cell lines	Inhibition of cell proliferation and decrease the expression of COX-2	[92]
*Withania somnifera* leaf extract	Leaves	Human glioma cell line (YKG1, U118MG and A172)	Inhibition of cell proliferation and increased the expression of NCAM and mortalin	[99]
*Withania somnifera* leaf extract and withaferin	Root	Human normal fibroblasts (TIG-3), breast carcinoma (MCF7), colon carcinoma (HCT116)	Increased DNA damage, oxidative stress, and downregulation of TPX2, TFAP2A, LHX3, and ING1	[100]
*Withania somnifera* root extract	Root	Human prostate cancer cells (LNCaP and 22Rv1)	Inhibition of cell proliferation, fatty acid synthesis and downregulation of the expression of c-Myc and p-Akt ^(S473^)	[101]
*Withania somnifera* root extract	Root	Prostate cancer cells (PC3)	Inhibition of cell proliferation, cell cycle arrest in G2/M phase and downregulation of the expression of IL-8 and COX-2	[102]
*Withania somnifera* roots extract and cisplatin	Root	Breast (MDA-MB-231) and colon (HT-29) cancer cells	Inhibition of cell proliferation, increased mitochondrial dysfunction, and ROS	[103]

NR: (Not reported) extractions sources or purchased from company.

### 2.3. Pharmacokinetic Studies and Bioavailability of Withania somnifera

The major drawbacks of biological agents are rapid metabolism, quick excretion, and poor bioavailability, which reduce their potential as anticancer agents [104]. It is critical to know a drug’s bioavailability before recommending it to treat a disease. WS bioavailability has been shown in preclinical tests to be acceptable [105,106]. In a recent assessment, the ideal oral pharmacokinetics of Withaferin A was determined in male rats and the in vitro screening of absorption factors by liquid chromatography–mass spectrometry (LC–MS/MS) and quadrupole trap mass spectrometry (Q-TRAP) analysis [105]. Male rats were given Withaferin A intravenously (5 mg/kg) and orally (10 mg/kg), and the oral bioavailability was found to be 32.4  ±  4.8%. Furthermore, in vitro findings revealed that Withaferin A was readily transported through Caco-2 cells and Withaferin A did not seem to be a substrate for P-glycoprotein. 

The stability of Withaferin A in male rat or human intestinal microflora was assessed as drugs given orally always interact with a significant population of intestinal microflora in the digestive tract and Withaferin A is susceptible to bacterial degradation. There were no significant differences in the stability of Withaferin A in male rats and humans when tested in formulated gastric fluid (stable), in intestinal microflora solution (gradual reduction), and in liver microsomes (swift expenditure with a half-life of 5.6 min). As a result, the initial metabolism of Withaferin A was confirmed using rat intestine-liver in situ perfusion, which showed that Withaferin A quickly dropped and remained at 27.1% in the first hour, while the level of the three key metabolites (M1, M4, and M5) detected by Q-TRAP analysis increased. 

Patil et al. [106] identified Withaferin A and Withanolide A in mouse plasma using high-performance liquid chromatography-tandem mass spectrometry. In this study, plasma samples were pretreated with tert-butyl methyl ether and the simple liquid–liquid extraction method was performed. Here, a Hypurity C18 column using methanol and ammonium acetate (95:5, *v*/*v*) is used as a mobile phase to partition the analytes and identified by electrospray ionization in the multiple reaction monitoring mode. The mass transition ion-pair was *m*/*z* 437.2 →292.2 for tianeptine (IS); *m*/*z* 471.3 → 281.2 for Withaferin A; and *m*/*z* 315 → 9270 for clonazepam (IS) and *m*/*z* 488.3 → 263.1 for Withanolide A. Furthermore, this technique demonstrated good linearity (r2 > 0.997) across the concentration dosage of 0.476–116.050 ng/mL for Withanolide A and of 0.484–117.880 ng/mL for Withaferin A. The lower bounds of quantification (LLOQs) for Withanolide A and Withaferin A were determined to be 0.476 ng/mL and 0.484 ng/mL, respectively, which is less than the C_max_/20 ratio, indicating that the technique has sufficient sensitivity to detect these withanolides in plasma samples. The optimum precision (% CV) and accuracy (% bias) were recorded between 3.7–14.3% and −14.4–4.0%, respectively. This proven technique was effectively used in pharmacokinetic research to estimate Withaferin A and Withanolide A in mice plasma after oral dosage of WS root aqueous extract. 

**Figure 2:**
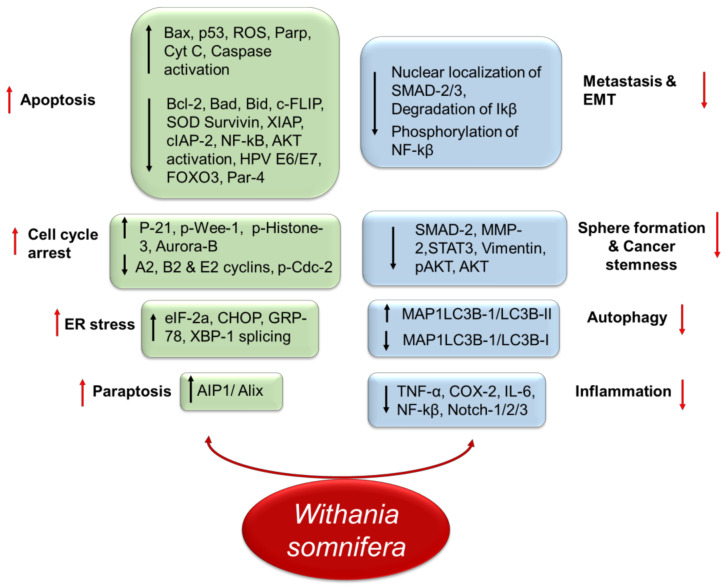
Various molecular targets of *Withania somnifera* in cancer cells. WS influence the apoptosis, cell cycle, ER stress, and paraptosis while involving reducing metastasis, EMT, stemness, autophagy, and inflammation.

The withanolides are rapidly absorbed after oral administration and withaferin A is relatively nearly twice as bioavailable as Withanolide A [106]. WS has been documented to have efficient pharmacological activities, including anticancer activity in vitro and in vivo. The WS can selectively target and kill cancer cells by activating various apoptosis-related molecular and cellular pathways. Various findings on the antitumor potential of WS revealed its regulatory impact on various erratic signaling pathways implicated in cancer establishment and progression, such as NF-κB, COX-2 and phosphatidylinositol 3-kinase/protein kinase B (PI3K/Akt) Figure 2. The in vitro cytotoxicity and targeted and regulatory aberrant mechanisms of WS are summarized in Table 2.

## 3. Role of *Withania somnifera* in Cancer

### 3.1. Lung Cancer

Lung cancer is the predominant cause of cancer-associated mortalities globally [1]. A combination therapy of paclitaxel and WS (400 mg/kg body weight) extracts in treating benzo(a)pyrene-induced lung carcinogenesis in Swiss albino mice shielded the mice from reactive ROS-induced damage through antioxidant activity, restored immune activity, and decreased cell viability [107,108]. It has also been shown that Withaferin A suppressed the binding of U937 monocytic cells with A549 cells stimulated by tumor necrosis factor-α (TNF-α) via deregulation of vascular cell adhesion molecule 1 and intracellular adhesion molecule 1 expression, blockade of Akt phosphorylation, and shunted nuclear factor kappa B (NF-κB) activity [25]. Additionally, Withaferin A has demonstrated potent activity against TNF-α-induced epithelial–mesenchymal transition (EMT) and TGF-β in A549 and H1299 non-small cell lung cancer (NSCLC) cell lines and has also induced apoptosis and cell cycle arrest by inhibiting the PI3K/Akt pathway [109,110]. These findings encourage the testing of efficacy of WS as a single agent or with other chemotherapeutic agents for lung cancer therapy.

### 3.2. Breast Cancer

Breast cancer is the leading form of cancer in females [1,111]. The heterogeneity of breast malignancies greatly influences their aggressiveness and ability to naturally metastasize [112]. Fluorescence microscopy studies on breast cancer cell lines demonstrated the efficacy of Withaferin A in initiating mitotic arrest in MDA-MB-231 and MCF-7 cell lines as well as the Ser10 residue-targeted phosphorylation of H3 histone [113]. Five-week intraperitoneal supplementation of 4 mg WA/kg body weight in female nude mice injected with MDA-MB-231 cells exhibited significantly reduced tumor growth while there was Bim-dependent and FOXO3a-induced apoptosis in the same cells in in vitro [20]. Furthermore, a recent study has evinced a novel apoptosis induction mechanism using Withaferin A, in which levels of survivin proteins, the cellular inhibitor of apoptosis-2 (cIAP-2), and the X-linked inhibitor of apoptosis (XIAP) all decreased after about 6 h of WA treatment; in in vivo, only survivin proteins were inhibited by Withaferin A [114]. Withaferin A has been shown to inhibit oxidative phosphorylation in breast tumors and induce the apoptotic death of cells through ROS [115]. 

Thaiparambil et al. [56] demonstrated the ability of Withaferin A to elevate phosphorylation of vimentin at its Ser56 residue, which indicates disassembly of vimentin, and thereby enhanced anti-metastatic and anti-invasive phenotypes in in vitro and in vivo. Withaferin A can potentially augment the distinct Cys328 vimentin residue in Human Umbilical Vein Endothelial Cells (HUVECs) covalently, resulting in vimentin denigration in in vivo and inhibition of neovascularization [116]. Furthermore, Withania root extracts have been shown to influence the EMT in breast cancer in in vitro and in xenograft mouse models supplemented with MDA-MB-231 cells [117,118]. The immunohistochemistry studies demonstrated that methylnitrosurea-induced mammary malignancies in female Sprague-Dawley rats caused a reduction in the proliferating cell nuclear antigen marker and Ki67 expression after Withaferin A root extract treatment [119]. Interestingly, transfection of MDA-MB-231 cells with ER-α inhibited Withaferin A-induced apoptosis but failed to obstruct the Withaferin A-induced cell cycle arrest at the G2/M phase. Withaferin A demonstrated anti-estrogenic tendencies by halting the growth of the estrogen receptor (ER)-positive T47D and MCF7 cells [120]. 

There have also been reports of anti-proliferative propensities of Withaferin A under various experimental settings [121]. For instance, Withaferin A suppressed the phosphorylation of Jak2 and signal transducer and activator of transcription-3 (STAT-3) [122]; inhibited NF-κB [123]; activated Notch-2 and Notch-4 upregulated [124]; and induced the overexpression of Elk-1-mediated Death Receptor five [125]. Research on the epigenetic properties of Withaferin A elucidated the potential of Withaferin A to methylate or demethylate numerous genes implicated in Triple-Negative Breast Cancer and inhibit their specific features of mildly aggressive luminal breast cancer with enhanced therapeutic sensitivity and response [126]. In conclusion, these studies highlight the exceptional therapeutic potential of active constituents of WS against breast cancer through anti-proliferative and anti-invasive molecular modes of action.

### 3.3. Prostate Cancer

Prostate cancer is the second most frequent cancer in men globally and accounts for 3.8% of cancer-related mortalities in men worldwide [1,111]. The anticancer activity of WS constituents has been fairly documented, such as the induction of prostate apoptosis response-4 (Par-4) dependent apoptosis in prostate cancer cell lines and regression of PC-3 xenografts in nude mice after a combination treatment of Withaferin A and other anti-androgens [73]. Furthermore, Withaferin A resulted in a dose-dependent inhibition of cell viability and led to the accumulation of Weal in the G2/M phase of the cell cycle [127] and facilitated vimentin denigration, which has been previously documented in breast malignancies, stimulated ROS generation, and decreased c-FLIP levels [56,81].

A 3-azido derivative of WA (3-azidoWA) restricted the cell invasion, mediated by extracellular Par-4-dependent inhibition of matrix metalloproteinase-2 (MMP-2) in PC-3 and HeLa cells [93]. Additionally, in vivo studies demonstrated a decreased expression of p-ERK and p-Akt and inhibited angiogenesis in mice [93]. 3-azidoWA also exhibited anticancer potential against prostate cancer by stimulating ER stress and improved chemosensitivity by influencing the shift from autophagy to apoptotic death in prostate cancer cells. Recent reports have shown the ability of WS root extracts to suppress lipogenesis in 22Rv1 cells, most likely by decreasing the expression levels of p-Akt and c-Myc, and this possibly indicates the mechanism of fatty acid metabolism in malignant cells and a novel strategy of inducing antitumor activity in prostate cancer [101].Kunimasa et al. [128] found that Withaferin A combined with glucose metabolism focused therapy might be an effective treatment for cancer cells resistant to tyrosine kinase inhibitors (TKI). Drug tolerance per sisters (DTPs) were formed in EGFR mutant lung cancer cell lines were treated with gefitinib and characterized by increased senescence (CD133 low) and stemness (marked by CD133 high population). Senescent cells exhibit the SASP (senescence-associated secretory phenotype) phenotype and may connect with other cells through secreted substances that have been SASP treated with gefitinib conditioned medium enhanced CD133 high in CSCs. The researchers recommended combining glucose metabolism targeting treatment with Withaferin A to target CSCs (as senescent CD133 low cells have enhanced glucose metabolism).

### 3.4. Colon Cancer

Colon cancer ranks third globally incidence wise and ranks second in mortality cases among the various cancers [1,111]. In Swiss albino mice, it was observed that ethanolic extracts of WS evinced immunoregulatory tendencies in azoxymethane-induced colon cancer [129]. Withaferin A has also demonstrated anticancer activity against colon cancer by targeting and downregulating Notch-1 signaling via targets such as Hey-1 and Hes-1 and concurrently suppressing crosstalk between Notch-1 and Akt/mTOR pathways. This makes the Notch-Akt-mTOR axis an attractive therapeutic target in colon cancer therapy [130]. Furthermore, there was observed dose-dependent apoptotic induction in three colon cancer cell lines as seen by the upregulated expression of apoptotic markers such as Poly ADP ribose polymerase (PARP) and caspase-3, as well as upregulated phosphorylation of c-Jun and JNK [130]. Moreover, Withaferin A caused cell cycle arrest at the G2/M phase of the cell cycle as a result of spindle assembly checkpoint blockade that invariably results in mitotic disruption, and proteasomal denigration of Mad2 and Cdc20, which ultimately results in chromosomal instability [82]. 

Other reports indicate that Withaferin A is capable of inhibiting migration and the IL-6 mediated the stimulation of STAT-3 in HCT116 cells [131]. Additionally, Withaferin A treatment of HCT116 xenograft tumors in Balb/c nude mice showed a pronounced decrease in tumor weight and volume [131]. A drastic reduction in tumor progression, volume, polyp size, and adenomas in Withaferin A-treated mice relative to controls highlights the need for active investigation into clinical application of Withaferin A [24]. A recent report indicates a combination regimen of 5-fluorouracil with Withaferin A inhibited colorectal cancer cell viability and stimulated the ER-stress-mediated induction of apoptotic cell death and autophagy, while causing cell cycle arrest at the G2/M phase [132]. Additionally, the induction of apoptosis was mediated by the PERK axis of ER stress and was non-toxic to healthy colon cancer cells [132].

### 3.5. Leukemia

Withaferin A has demonstrated potent anticancer activity against solid tumors. In this section, its efficiency in inhibiting hematological malignancies would be discussed. Initial research reports demonstrated that Withaferin A-mediated inhibition of cell proliferation in several malignant lymphoid and myeloid cells, cell cycle arrest at the sub-G0 phase, and apoptotic induction via the p38/MAPK signaling pathway [71]. Furthermore, L-Asparaginase isolated and purified from WS fruit demonstrated anti-proliferative tendencies in acute lymphoblastic leukemia cells obtained from leukemia patients [87]. Conclusive studies on leukemia cell lines such as U937 and others directly correlated Withaferin A with improved ionizing radiation-mediated cell death via ROS stimulation, cell cycle disruption at the G2/M phase and simultaneous upregulation of JNK signaling, and deregulated Akt phosphorylation [57,133,134]. Moreover, WS has demonstrated various anti-leukemic tendencies such as the ability of WS root extracts to improve ROS generation, induce cell cycle arrest, pump intracellular calcium, and denigrate DNA structure of T-lymphoblastoid cell lines [135].

### 3.6. Other Cancers

The anticancer activity of WS has been fairly documented in other carcinomas. In melanoma, it was observed that Withaferin A augured ROS production that resulted in mitochondrial-mediated cell death in melanoma cells with a range of IC_50_ values between 1.8 to 6.1 mM and caused DNA damage. In Swiss albino mice models, WS root extracts therapy caused pronounced weight gain and decreased skin lesions [136]. Also, in human osteosarcoma cells and gastric and oral cancer cells, WA led to the G2/M phase cell cycle arrest [137,138,139]. However, there is a paucity of comprehensive research of antitumor effects of Withaferin A in such cancers, though in oral cancer, studies have identified selective degradation of oral cancer cells as a result of oxidative stress and depolarized mitochondrial membrane potential as well as DNA fragmentation [137]. 

In pancreatic cancer (PanCa), a combination regimen of Withaferin A and oxaliplatin resulted in the intracellular accumulation of ROS, which correlated with downregulation of Akt and apoptotic cell death. This provided the strongest evidence yet of effective antitumor activity of a combination therapy of Withaferin A and oxaliplatin in PanCa therapy [140]. Similar evidence was seen in a combination therapy of doxorubicin and Withaferin A in ovarian cancer which led to improved ROS generation and stimulated autophagy [141]. In vivo studies in mice showed a 70–80% reduction in tumor mass upon combination treatment of Withaferin A and doxorubicin relative to the control or single drug treatments [141]. 

Another study demonstrated therapeutic efficacy of Withaferin A alone or Withaferin A plus cisplatin in downregulating Notch-1 signaling and repressing metastasis in nude mice [142]. Immunohistochemistry (IHC) and proteomic analysis showed pronounced decrease in cancer stem cell biomarkers (CCD117, CD34, Oct-4, CD44, and CD-24) and metastatic biomarkers such as Notch-1. Interestingly, the administration of cisplatin alone in xenograft mice had the opposite effect, highlighting the efficacy of the Withaferin A-cisplatin combination treatment regimen in drug-resistant ovarian cancer [142]. Withaferin A-treatment of SKOV3 and CaOV3 cells attenuated cell viability and clonogenicity, initiated apoptosis, and caused cell cycle arrest at the G2/M phase [55].

Withaferin A demonstrated potent anti-proliferative effects in cervical cancer cells as documented by Munagala et al. [67]. These studies showed that Withaferin A suppressed most tumor characteristics including CaSki cell viability (IC50 = 0.45 mM), deregulated HPV E6/E7 oncoproteins, decreased STAT-3 phosphorylation, and upregulated p21 and p53 proteins [143]. In vivo studies in athymic nude mice also yielded a pronounced decrease in tumor mass [67]. A dose-dependent inhibition of TGF-β induced Akt phosphorylation and decreased MMP2 and MMP9 expression in Withaferin A-treated CaSki and SK-Hep1 cells, indicating anti-invasiveness of WA [143]. Withaferin A has demonstrated potency against renal carcinoma as well. Withaferin A induced apoptosis in CaSki cells, suppressed JAK-2 activation, IL-6-mediated STAT-3, Akt, and Bcl-2 phosphorylation, and upregulated the expression of glucose-regulated protein (GRP)-78 and CAAT/enhancer-binding protein-homologous protein as well as the stimulation of ROS-dependent expression of the endoplasmic reticulum (ER) stress markers such as the phosphorylation of eukaryotic initiation factor-2α and X-box binding protein 1 (XBP1) splicing [21,144,145]. Interaction between Withaferin A and the Cys179 residue found in the catalytic site of IKKβ also suppressed NF-κB activity in HEK293T cells [146].

### 3.7. Chemosensitization and Synergistic Actions of Withania somnifera

The development of resistance to standard chemotherapy indicates that single drugs may not be enough for cancer treatment. Combination therapy has received attention in recent years as a novel cancer treatment approach [147]. Traditional chemotherapeutics combined with therapy with phytochemicals like WS may enhance the therapeutic effectiveness in cancer treatment. WS has a chemo-sensitizing impact on many cancer types by altering numerous signaling pathways, such as MAPK/ERK, PI3K/AKT, and NF-κB. Interestingly, many in vitro and in vivo investigations have shown the therapeutic potential of WS as a combinatorial anticancer medicine. In this section, we discuss the synergistic effects of co-treatment with chemotherapeutic agents and WS in inhibiting various carcinogenic pathways. Chemo-sensitization and synergistic effects of WS are thought to enhance intracellular concentrations of chemotherapeutic drug(s) in cancer cells as well as at the tumor site. 

Kyakulaga et al. [148] have shown synergistic effects of paclitaxel and Withaferin A against human NSCLC. In H1299 and A549 cells, paclitaxel, and WA co-treatment reduced cell proliferation, colony formation, migration, invasion, and enhanced apoptosis. Contrary to expectations, the synergy of paclitaxel and Withaferin A was increased when cells were pretreated with Withaferin A, suggesting an anti-chemosensitivity effect. On the other hand, Withaferin A inhibited both paclitaxel-susceptible (TS-A549) and paclitaxel-resistant (TR-A549) cells in in vitro and in vivo. Withaferin A suppresses NSCLC cell growth via oxidizing thiols. In doxorubicin-sensitive K562 and doxorubicin-resistant K562/Adr cells, Withaferin A alone can negate attenuated caspase activation and apoptosis, while quercetin-mediated caspase regulation and apoptosis is just delayed. However, only Withaferin A lowers intracellular protein levels of Bcl2, Bim, and *p*-Bad, while increasing PARP cleavage, caspase 3 activation, and apoptosis, perhaps through thiol oxidation [149]. Withanolide D (C4β-C5β,C6β-epoxy-1-oxo-,20β, dihydroxy-20S,22R-witha-2,24-dienolide; Withanolide D) is isolated from WS initiates leukemic apoptosis by the upregulating activation of neutral sphingomyelinase-ceramide cascade, facilitated by synergistic activation of c-Jun N-terminal kinase and p38 mitogen-activated protein kinase [97]. This study shows that Withanolide D may raise ceramide levels in myeloid (K562) and lymphoid (MOLT-4) cells and enhance JNK and p38MAPK phosphorylation downstream of ceramide. In addition, N-SMase 2 is a major mediator of Withanolide D-induced apoptosis, and N-SMase 2 siRNA and N-SMase inhibitor (GW4869) reduced Withanolide D-induced ceramide production and MKK4 and MKK3/6 phosphorylation but not MKK7 in leukemic cells. The inhibitor GW4869 also protected these cells against Withanolide D-mediated mortality and reduced apoptosis, while Fumonisin B1, a ceramide synthase inhibitor, had no impact. Also, Withanolide D efficiently triggered apoptosis in newly separated lymphoblasts from patients, through JNK and p38MAPK activation [97].

In prostate cancer cells, PAWR-regulated the suppression of Bcl-2 influences shift from 3-azido Withaferin A induced autophagy to apoptosis [150]. As a result, many MAP1LC3B and EGFP-LC3B puncta accumulated, and SQSTM1 gradually degraded. Higher toxic doses of 3-azido Withaferin A increased CaP cell ER stress, resulting in the activation of apoptosis by increasing PAWR expression, which inhibited Bcl2 and BECN1l expression, both of which are involved in autophagy. Overexpressed PAWR inhibits BECN1 in CaP cells, causing the Bcl2-BECN1 connection to be disrupted. Furthermore, with the lethal concentrations of 3-azido Withaferin, pawr-KO MEFs showed extensive autophagy signals, demonstrating the importance of PAWR in the transition from autophagy to apoptosis. Finally, overexpression of EGFP-LC3B and DS-Red-BECN1 in CaP cells resulted in a delay in the apoptotic turnover at greater 3-AWA concentrations. Another benefit was that it increased chemosensitivity by making prostate cancer cells more susceptible to apoptosis, which is why it has therapeutic promise [150]. 

Furthermore, sub-toxic concentrations of 3-azido Withaferin A suppressed cancer cell motility and invasion in wound healing and the Boyden chamber invasion by inhibiting MMP-2 activity in gelatin zymography, which is a significant barrier in chemo-sensitivity. An external activation of the tumor suppressor candidate Par-4 protein by 3-azido Withaferin A generated a new mechanism, and an immunoblot analysis revealed an associated significant decrease in pAkt/pERK signaling. This study also found that 3-azido Withaferin A inhibited MMP-2 through secretory Par-4, which is consistent with our zymography findings. MMP-2 gelatinase activity was not restored by 3-azido Withaferin A apoptotic suppression. In addition, 3-azido Withaferin A inhibited neovascularization in mice using the Matrigel plug test when administered in a dose-dependent manner [93].

**Table 2:** In vitro cytotoxic activity and targeted molecular mechanisms of *Withania somnifera* in different cancer types.

Cancer	Cell Line	Targeted Molecular Mechanisms	Ref.
Lung cancer	A549	Cell cycle arrest ↑; PI3K/Akt pathway↓	[109]
	H1299 and A549	TGF-β and TNF-α induced EMT ↓; nuclear translocation of Smad 2/3 and NF-κB ↓	[110]
	H1299, CL141, CL149, and A549	ROS, autophagy, and apoptosis ↑; mTOR/STAT3 signaling ↓	[79]
Breast cancer	MCF7 and MDA-MB-231	G2/M phase cell cycle arrest ↑; ROS generation and apoptosis ↑; ER-a, XIAP, cIAP-2 and survivin ↓	[19,20]
	MCF7 and MDA-MB-231	Cell migration, EMT and invasion ↓; IL6 induced STAT3 activation ↓; Notch2 and Notch4 ↑; mitochondrial membrane potential ↓	[100,122,125]
	SUM-159 and MCF-7	Mammosphere formation ↓, ALDH1 activity ↓, bCSCs↓;	[23]
Glioblastomas	GL26, U251, and U87	Cell proliferation ↓; G2/M phase cell cycle arrest ↑; ROS generation ↑; Akt/mTOR and MAPK pathway ↓	[151,152]
Microglial	BV2	Nrf-2 and HO-1 ↑; filopodia formation ↓	[153]
Neuroblastomas	IMR-32, U87-MG, C6, GBM39, and GBM2	Cell proliferation ↓; G0/G1 cell cycle arrest ↑; Cyclin D1 ↓; p-Akt, PSA-NCAM, Bcl-xL, MMP-2, MMP-9 ↓	[69,154,155]
Oral	CAL27 and Ca9-22	Cell proliferation ↓; G1 phase cell cycle arrest ↑; ROS generation, DNA damage and mitochondrial membrane depolarization ↑	[137]
Osteosarcoma	U2OS and MG-63	Cell proliferation ↓; G2/M phase cell cycle arrest ↑; cyclin B1, cyclin A ↓; p-Chk1, p-Chk2 ↑	[138]
Leukemia	THP-1, HL-60, MDS-L, and Ramos	Apoptosis↑; G2/M phase cell cycle arrest ↑; ROS ↑	[134,135]
Prostate	DU 145 and PC3,	Cell proliferation ↓; G2/M Phase cell cycle arrest ↑; ROS and autophagy ↑	[81,127]
Ovarian cancer	CaOV3, SKOV3, and A2780	Cell proliferation ↓; apoptosis ↑; ROS ↑; G2/M cell cycle arrest↑; Notch1, Notch2, otch3, Bcl-2, Akt ↓	[141,142]
Melanoma	Lu1205, M14, Mel501, and SK28	Cell viability ↓; apoptosis ↑; ROS↑; DNA fragmentation and mitochondrial membrane depolarization ↑	[78]
Gastric cancer	AGS	Cell viability ↓; Apoptosis ↑; G2/M cell cycle arrest ↑; ROS ↑; Cell migration and invasion ↓	[139]
Gastrointestinal	UP-LN1	Apoptosis ↑; CXCR4/CXCL12 and STAT3/IL-6 axis ↓	[156]
Thyroid cancers	SW1736 and BCPAP	BRAF, Raf-1 and, ERK ↓; cell cycle arrest at G2/M phase ↑	[157]

Symbols: ↑, increased or up-regulated; ↓, decreased or down-regulated.

### 3.8. Clinical Trials

WS actively inhibits a variety of oncogenic signaling molecules and warrants further clinical investigations. There is, however, a dearth of studies in this field that is centered on cancer, with only three clinical cancer-related investigations out of 11 total studies on WS. There are about 29 clinical trial studies reported on clinicaltrials.gov (https://clinicaltrials.gov/ct2/results?cond=&term=Withania+somniera&cntry=&state=&city=&dist= accessed on 3 March 2022). In an open-access nonrandomized comparative study on 100 patients with breast cancer; WS root extract was given to patients in the study group at a dose of 2 g every 8 h throughout the duration of chemotherapeutic treatment. It was observed that WS root extract exhibited therapeutic potential against cancer-related fatigue and improved the quality of life [54]. In another study, 24 participants were recruited to assess the efficacy of curcumin formulation and Ashwagandha extracts on advanced osteosarcoma. The conclusion of this study, however, has not been properly stated [158].

A prospective, randomized double-blind, placebo-controlled study conducted by Chandrasekhar et al. [32] evaluated the pharmacological profile of a highly concentrated full spectrum isolate of Ashwagandha roots in decreasing stress-induced anxiety and ameliorating the health of stressed participants. The extract was found to be safe, tolerable, and effective at decreasing stress and anxiety. However, another study reported the WS-mediated improvement of NK cell activity after the consumption of tea infused with WS-active herbal drugs [159]. Zwickey et al. [160] also investigated the effects of Ashwagandha on stress, inflammation, and immune cell activation in 25 participants. The root extract may be utilized as an adjuvant treatment in cancer patients to alleviate stress and anxiety. Table 3 outlines Withaferin A clinical studies on different disorders. Further studies are required to examine Withaferin A’s therapeutic potential in cancer. A randomized, double-blind, placebo-controlled study by Chengappa et al. [161] demonstrated the benefits of WS, including its safety in patients with recent schizophrenic inflammation.

### 3.9. Immunomodulatory Activity and Hematopoiesis Actions of Withania somnifera

*Withania somnifera* is an immunostimulant herbal remedy that is used to boost general health and prevent illness in the elderly [15,162,163,164]. Toll-like receptors, transcription factors, and inflammasomes all have a role in regulating inflammatory cytokines and chemokines [165]. NF-κB is the most researched transcription factor for modulating inflammatory cytokines in a variety of cell types [166]. Constitutive NF-κB activation has been demonstrated to increase the expression of NF-κB-related genes such as inflammatory cytokines/chemokines like CCL20/MIP-3 and granulocyte-macrophage colony stimulating factor (GM-CSF) [167]. WA’s ability to suppress NF-κB has been investigated in a variety of cell types and with a variety of triggering events [168]. A recent study by Kaileh et al. [169] showed that WA suppressed NF-κB activation by directly inhibiting IKKβ activity through thioalkylation, which are steroid lactones produced from WS, such as Withanolide A and 12-deoxywithastramonolide, are significantly less potent. The administration of a methanolic extract of the WS plant roots (1–256 g/mL) to mice macrophages resulted in an increase in nitric oxide generation due to nitric oxide synthase activation. 

A recent study shows that WS has significant cytotoxic and cytostatic potential and induced immunogenic cell death (ICD) in human T leukemia cells [135]. In in vitro, WA inhibited mitogen-induced T-cell and B-cell growth without causing cell death. The upregulations of activation markers on T-cells (CD25), B-cells (CD80, CD86, and MHC-II), and the generation of Th1 and Th2 cytokines were also inhibited by WA. In microglial cells, WA reduced LPS-induced COX-2 and prostaglandin E-2 (PGE2) synthesis, while inhibiting TNF-α and IL-1β production in mononuclear cells [170,171]. The immunomodulatory effects of an alcoholic extract of the plant roots were studied in mice using cyclophosphamide, azathioprine, or prednisolone myelosuppression models. The extract enhanced the quantity of blood cells, the cellularity of bone marrow, and the number of α-esterase positive cells [172,173,174]. A study reports that Withaferin A suppressed iNOS expression and nitric oxide generation by Akt activations and downregulated LPS-induced NF-κB in RAW264.7 cells [175]. Dubey et al. show that Withaferin A treatment of THP-1 cells prevents NF-kB from translocating to the nucleus, resulting in lower levels of cytokine release [27]. Withaferin A inhibits caspase-1 activation by altering the nigericin-induced co-localization of NLRP3 and ASC proteins [27].

## 4. Nanotechnology-Based Strategies for the Delivery of WS

Multiple in vitro and in vivo studies have shown that WS exhibits anticancer properties. However, due to its poor water solubility, poor biodistribution, and multi-targeting capability, it may cause inevitable systemic toxicity. Nanotechnology approaches may help to reduce such uninvited adverse effects and improve clinical translation. Nanotechnology has gained a lot of attention in recent times because of its improved payload delivery to specific therapeutic locations, as well as its potential to alter cellular permeability, absorption, and pharmacokinetic profiles [176,177,178,179]. In recent years, the bioinspired production of NPs employing various biological systems, such as microbes and plants, has gained prominence [180,181,182,183]. Plant-based NPs production has attracted attention because of plant availability, tolerance, and eco-friendly NPs synthesis [180,181,182,183]. The phytoconstituents in the extract reduce and stabilize the generation of non-toxic NPs [180,181,182,183]. Among the many nanoparticle carriers (iron oxide, silicone material, and quantum dots), AuNPs are preferred owing to their high biocompatibility, quenching efficiency, ease of production, numerous functions, and adjustable optical nature [184,185,186]. According to Grand View Research, Inc., the nanomedicine industry is expected to be valued at USD 350.8 billion by 2025 [187]. 

**Figure 3:**
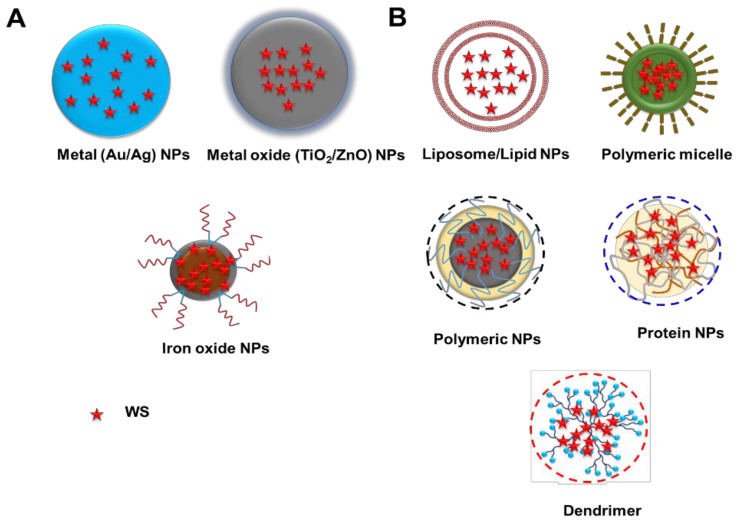
Different type of *Withania somnifera* nanoparticles. (**A**) inorganic nanoparticles and (**B**) organic nanoparticles. Red stars represent *Withania somnifera*.

Nanomedicines are divided into two categories (Figure 3): organic nanoparticles (such as polymeric, liposomes, etc.) and micelles and inorganic nanoparticles (such as gold, silica, and iron oxide, etc.).

Inorganic nanoparticles have been utilized in several applications, including lymph node imaging, hyperthermia, and anemia therapy, and a section of them have been successful in preclinical research and clinical studies. Organic-based nanoparticles, like lipid and polymer nanoparticles, have successfully entered the clinical phase and are now available on the market for a variety of applications such as immunization, microbial infection, and cancer.

### 4.1. Inorganic Nanoparticles

Various inorganic nanomaterials utilized in bioimaging and therapeutics include metals, metal oxides, semiconductors, and lanthanide-laced NPs [188,189,190,191,192,193,194]. In bioimaging, inorganic NPs have been used as imaging probes that improve imaging methods like as computed tomography, magnetic resonance imaging, and optical imaging owing to their magnetic, X-ray attenuation, and optical characteristics [195,196,197]. Inorganic NPs have demonstrated immense potential in cancer disease therapy as whole drugs or drug delivery systems [198,199,200,201]. Furthermore, smart inorganic nanotherapeutics, which are stimuli responsive and target specific, have been generated to yield precise cancer treatment [202,203,204]. Some inorganic NPs that have gained clinical approval for disease therapy include the iron oxide NPs, Dexferrum, Feraheme, Infed, Feridex, Ferrlecit, Venofer, and Nano-therm [205]. Metallic NPs have been synthesized using aqueous extracts of various plant parts such as seeds, roots, leaves, stems, and fruits.

#### 4.1.1. Gold Nanoparticles (AuNPs)

AuNPs offer unique qualities such as a huge surface area, the capacity to bind with different molecules, high stability, outstanding biocompatibility, and minimal toxicity, as well as the ability to control drug release [206,207]. AuNPs can easily be tagged with ligands for selective targeting because they can make bonds with amine and thiol groups. Because of their nano conjugation capacity, phytochemicals have been employed to develop Au nanocarrier-based conjugation for active-targeting drug delivery. AuNPs have been assessed for clinical applications as a result of their unique physicochemical properties. Tabassam et al. [208] recently assessed the anti-cancer potential of Withanolide-A with 20 nm AuNPs conjugates against SKBR3 breast cancer cell lines. The AuNPs can be generated by several in vitro and in situ methods, but only limited techniques can yield uniformly spherical particles. 

A chemical synthesis method was utilized in the preparation of spherical AuNPs as well as conjugation of 10 μg/mL of Withanolide-A (1) with spherical AuNP solution, based on the same principle [96], and the phytochemical gold nanoconjugates was assessed through various analytical techniques, UV-visible spectroscopy, dynamic light scattering (DLS), and transmission electron microscopy (TEM). The absorption peak of AuNPs was λmax at 523.5 nm, similar to results from previous findings. DLS data showed that the presence of a slightly positively charged single hydrogen atom on an Au surface changed the zeta potential value from −44.3 ± 0.86 mV before conjugation to −20 ±0.1 mV after conjugation. A slight variation of the PDI from 0.285 ± 0.02 to 0.3 ± 0.009, which was less than 0.3, indicates non-uniformity in aggregation and size of the particles. The hydrodynamic size increased from 25.35 ± 0.61 nm to 29.73 ± 0.65 nm, which indicates the attachment of a single molecule on Au surface [209]. TEM analysis of the size and distribution of synthesized AuNPs recorded an average size of 20 nm on micrograph before and after conjugation. Moreover, Au nanoconjugates with Withanolide-A efficiently inhibited the growth of SKBR3 cells at half maximal concentration in contrast to pure Withanolide-A [208].

#### 4.1.2. Titanium Oxide Nanoparticles (TiO_2_ NPs)

The phytomediated synthesis of TiO_2_ NPs has a lot of potential for killing bacteria, viruses, fungus, and cancer cells, and it can also be used to treat malignant tumors as a catalyzer [210,211]. Due to its extreme hydrophilicity, low toxicity, strong thermal conductivity, good optical absorption, and chemical and thermal durability in in vivo, TiO_2_ NPs could be a promising candidate for biomedical applications as agents in converting photon energy into heat in the PTT method [212,213]. The biomediated production of TiO_2_ NPs has been employed in disease therapy, surgical product manufacturing, photocatalysis, agriculture and tissue engineering [214,215]. 

Poly(ethylene glycol) (PEG) could be added to the surfaces of TiO_2_ NPs to improve their biocompatibility. Several plants and plant organs have been utilized in TiO_2_ NPs including roots, leaves, peel, flower, seeds, and pollen [216,217,218,219]. TiO_2_ NPs are synthesized by various methods such as chemical vapor deposition, hydrothermal and reversed micellar methods, and the sol-gel process [220,221,222,223]. Titanium dioxide is a high-quality photocatalysts [224,225,226] with a wide bandgap of 3.2 eV and frequently used in optoelectronic gadgets and dye-sensitized solar cells [227,228,229,230]. Al-Shabib et al. synthesized phytomediated green TiO_2_ NPs from *Withania somnifera* root extract, tested their broad-spectrum biofilm inhibitory efficacy against bacterial and fungal pathogens, and assessed HepG2 cytotoxicity [231]. The synthesized NPs significantly reduced the viability of HepG2 in in vitro and may be useful in the treatment of liver cancer. In another study, Maheswari and colleagues noted the antitumor and antibacterial properties of hydrothermally synthesized bio-modified TiO_2_ nanoparticles with WS on KB oral cancer cell lines [230]. Bio-modified TiO_2_ nanoparticles show dose-dependent activity in KB oral cancer cell lines. When compared with the modified bio-modified TiO_2_ nanoparticles, pure TiO_2_ nanoparticles had a higher viability percentage, proving that plant dopants treated with TiO_2_ are effective anticancer agents [230].

#### 4.1.3. Silver Nanoparticles (AgNPs)

AgNPs are among the most often used nanomaterials because of their antimicrobial characteristics, easily modified surface, controllable size and shape, strong electrical conductivity, and optical features [232]. AgNPs silver nanoparticles have been employed in a wide range of applications, including biosensors, electrical compounds, antimicrobials, and pharmaceuticals [233]. AgNPs can be synthesized through various methods such as physical (e.g., Turkevich), chemical (e.g., citrate or NaBH_4_), and biological methods (e.g., plants, fungi, algae and other organic sources) with remarkable stability [232,234]. Biodegradable compounds and polymers can be added to the surface of AgNPs to improve their biocompatibility. Alternatively, these nanoparticles can be integrated into hybrid systems [235]. 

Tripathi et al. developed AgNPs through the reduction of silver nitrate solution using an in vitro-produced leaf extract of *Withania coagulans* Dunal (WcAgNPs) and evaluated anticancer activity with SiHa cell lines) [236]. WcAgNPs had a size of 14 nm and a spherical form with a face-centered cubic structure. WcAgNPs have excellent in vitro cytotoxicity in cervical cancer cells SiHa and induced apoptosis at 48 hrs. Gaurav et al., synthesized silver nanoparticles with root extract of WS (AgNPs-REWS) and tested for anticancer activity in in vitro [237]. A stable AgNPs made from Rhodiola imbricata root extract (RIW) and WS (RIWS-AgNPs) have been shown to have prospective uses in biomedicine and agriculture as phytostimulant, antioxidant, and anticancer agents [238]. Furthermore, RIWS-AgNPs have potent cytotoxic action against the HepG2 cancer cell line in a dose-dependent manner (cell viability: 9.51 ± 1.55%) [238].

#### 4.1.4. Zinc Oxide Nanoparticles (ZnO NPs)

ZnO NPs are showing diverse medical applications and great promise in cancer treatment due to their high potency and selectivity for cancer cells [239,240]. As a possible substitute for photothermal therapy (PTT), zinc oxide (ZnO) has excellent chemical stability and minimal toxicity, as well as optical, electrical, and anticancer characteristics [241].The ROS and protein activity disequilibrium may be responsible for the cytotoxic action of ZnO NPs [242]. ZnO NPs are effective nanocarriers for the administration of several medications, such as DOX, paclitaxel, CUR, and baicalin as they have minimal toxicity and are biodegradable [242]. Kumar et al. show the immunomodulatory and protective effect of WS extract and Withaferin A supplementation on zinc oxide nanoparticles mediated the toxicity in a mouse model [243]. When ZnO NPs were delivered in vivo, a dose-dependent decrease in phagocytosis, an increase in NO generation, and an up-regulation of the TLR6 arginase gene were found to be significant. In the presence of WS and Withaferin A, however, ZnO NPs toxicity was reduced, with decreased TLR6 overexpression and restoration of phagocytic activity.

### 4.2. Organic Nanoparticles

Liposome-based nanomedicines employ drug encapsulation inside the phospholipid bilayer to improve its pharmacokinetics and biodistribution. Liposomes are globular vesicles surrounded by a phospholipid bilayer [244,245] and drug delivery mostly operates by passive targeting [205,246]. The advantages of liposomal drug delivery systems are as follows: (i) amphiphilic nature of liposomes enable them to deliver both hydrophobic and hydrophilic drugs; (ii) liposomal nanotherapeutics demonstrate enhanced accumulation and superior pharmacokinetics compared with non-liposomal agents at wound sites, resulting in decreased off-target toxicity and improved therapeutic efficacy; (iii) liposomes offer drug protection and stability and improves circulation half-life; and (iv) the functional coating of liposomal surfaces can yield targeted drug delivery systems [244,247,248,249,250,251]. However, the reticuloendothelial system (RES) and the mononuclear phagocytic network regulate liposomal clearance [252,253,254]. As such, PEG has been employed in liposomal modification to prolong the circulation half-life of liposomes [79,205,255,256]. 

Liposomal drug delivery systems are being assessed in clinical trials, including the liposomal nano formulations of docetaxel, paclitaxel, irinotecan, and cisplatin [257,258,259,260]. For instance, EndoTAG-1, a cationic liposome-based formulation of paclitaxel geared at PanCa therapy, liver metastases, and triple negative breast cancer has completed phase II clinical trials. Similarly, liposome-based nanomedicines of WS can be used for the cancer. Off-target toxicity and scale-up are the major obstacles in the way of the clinical translation of liposomal nano formulations. Recent liposomal formulations seek to improve precise disease targeting, such as ligand-functionalized liposomes, and have shown promising preclinical outcomes [261,262]. The disadvantages of polymeric micelles include minimal efficiency for intracellular drug release as well as possible off-target delivery. To navigate these limitations, stimuli-responsive polymeric micelles have been generated to facilitate sustained drug release in response to fluctuating environmental stimuli such as temperature or pH [263,264].

It is important to note that at the current time not all types of organic nanoparticles, such as self-assemblies, polymeric nanoparticles, dendrimers, and protein nanoparticles, have been utilized for the delivery of WS. However, future research may include the use of such clinically relevant carriers for efficient delivery of WS.

## 5. Future Perspective of WS Delivery

Phytochemicals have significant potential as anticancer agents. WS has anticancer properties through inhibiting pro-cancer mechanisms such as angiogenesis, migration, proliferation, invasion, and metastasis. WS promotes apoptosis through ROS generation, DNA damage, and regulation of oncogene and tumor suppressor gene expression. So far, there are no documented reports of toxicity of WS in humans. WS co-treatment may re-sensitize resistant cancer cells to chemotherapeutics and radiation. Despite several preclinical studies indicating WS cytotoxic potential against various cancers, physicians have not yet recognized its therapeutic value in treating cancer patients. Moreover, extant research on WS has barely evaluated its oral bioavailability, and investigation of these aspects of a new drug is essential before initiating clinical trials. Indeed, poor water solubility and biodistribution may restrict WS efficacy as an anticancer drug in somatic settings, and the dearth of extensive clinical investigations into WS as an anticancer agent may be due to these factors. 

Nano formulation of this compound may help overcome barriers associated with natural phytochemicals like WS. Because phytochemicals constitute a varied collection of substances, it is critical to tailor nanoparticle formulations to the desired therapeutic agents’ physical and chemical characteristics. Fortunately, nanoparticles may be constructed from different substances, including lipids and polymers. In animals, formulations may be rationally planned as well as experimentally optimized. Passive targeting is quite simple to implement and may be readily applied in clinical practice. Liposomal vincristine, liposomal paclitaxel, and paclitaxel polymeric micelles are current examples of available nano-formulations. Actively focused drug delivery systems can outperform passively targeted nanocarriers with EPR effects. Smaller systems like antibody-drug conjugates (ADC) fall under this category. The market has several ADCs and many more are in different phases of trials. Many variables influence active targeting, including receptor expression and systemic circulation accessibility. As a result, WS must be carefully designed and optimized in terms of formulation. Targeted nanoparticles, we believe, will be the next step in the therapeutic evolution of WS. In conclusion, further extensive preclinical and clinical research of WS is required to better understand and enhance its anticancer activity. We anticipate that a new generation of nanocarriers will significantly advance the clinical use of WS by using the rapidly developing expertise in this sector.

## Figures and Tables

**Table 3:** Clinical trials involving *Withania somnifera*) in Interventional Studies.

Conditions	Used Alone or in Combination	ClinicalTrials.gov Identifier	No. of Enrolled Patients	Outcome	Ref.
Breast Cancer	Root extract	NA	100	Improvement of quality of life and chemotherapy induced fatigue	[54]
Advanced Osteosarcoma	CUR formulation and Ashwagandha Extract	NCT00689195	24	Detailed is not available	[158]
Stress and Anxiety	Root extract	NA	64	Reduced stress and Anxiety improve self-assessed quality of life	[32]
NK Cell Activity	Polyherbal formulation	NA	32	Enhancement of NK cell activity	[159]
Stress, Inflammation, and Immune Cell Activation	3 mL of Ashwagandha for 5 days	NCT00817752	25	Detailed is not available	[160]
Schizophrenia	WS extract	NCT01793935	68	Significant benefit for people with schizophrenia exacerbation	[161]

## Data Availability

Not applicable.

## References

[1] Siegel R.L., Miller K.D., Fuchs H.E., Jemal A. (2021). Cancer Statistics, 2021. CA Cancer J. Clin..

[2] The Centers for Disease Control and Prevention, an Update on Cancer Deaths in the United States. https://www.cdc.gov/cancer/dcpc/research/update-on-cancer-deaths/index.htm.

[3] Banik K., Harsha C., Bordoloi D., Lalduhsaki Sailo B., Sethi G., Leong H.C., Arfuso F., Mishra S., Wang L., Kumar A.P. (2018). Therapeutic potential of gambogic acid, a caged xanthone, to target cancer. Cancer Lett..

[4] Kunnumakkara A.B., Banik K., Bordoloi D., Harsha C., Sailo B.L., Padmavathi G., Roy N.K., Gupta S.C., Aggarwal B.B. (2018). Googling the Guggul (Commiphora and Boswellia) for Prevention of Chronic Diseases. Front. Pharmacol..

[5] Manu K.A., Shanmugam M.K., Ramachandran L., Li F., Siveen K.S., Chinnathambi A., Zayed M.E., Alharbi S.A., Arfuso F., Kumar A.P. (2015). Isorhamnetin augments the anti-tumor effect of capecitabine through the negative regulation of NF-κB signaling cascade in gastric cancer. Cancer Lett..

[6] Manu K.A., Shanmugam M.K., Ramachandran L., Li F., Fong C.W., Kumar A.P., Tan P., Sethi G. (2012). First evidence that γ-tocotrienol inhibits the growth of human gastric cancer and chemosensitizes it to capecitabine in a xenograft mouse model through the modulation of NF-κB pathway. Clin. Cancer Res..

[7] Seca A.M.L., Pinto D. (2018). Plant Secondary Metabolites as Anticancer Agents: Successes in Clinical Trials and Therapeutic Application. Int. J. Mol. Sci..

[8] Shanmugam M.K., Lee J.H., Chai E.Z., Kanchi M.M., Kar S., Arfuso F., Dharmarajan A., Kumar A.P., Ramar P.S., Looi C.Y. (2016). Cancer prevention and therapy through the modulation of transcription factors by bioactive natural compounds. Semin. Cancer Biol..

[9] Patel S.M., Nagulapalli Venkata K.C., Bhattacharyya P., Sethi G., Bishayee A. (2016). Potential of neem (*Azadirachta indica* L.) for prevention and treatment of oncologic diseases. Semin. Cancer Biol..

[10] Singh N., Bhalla M., de Jager P., Gilca M. (2011). An overview on ashwagandha: A Rasayana (rejuvenator) of Ayurveda. Afr. J. Tradit. Complement. Altern. Med..

[11] Dhuley J.N. (1998). Effect of ashwagandha on lipid peroxidation in stress-induced animals. J. Ethnopharmacol..

[12] Mehta V., Chander H., Munshi A. (2021). Mechanisms of Anti-Tumor Activity of *Withania somnifera* (Ashwagandha). Nutr. Cancer.

[13] Rai M., Jogee P.S., Agarkar G., dos Santos C.A. (2016). Anticancer activities of *Withania somnifera*: Current research, formulations, and future perspectives. Pharm. Biol..

[14] Dutta R., Khalil R., Green R., Mohapatra S.S., Mohapatra S. (2019). *Withania somnifera* (Ashwagandha) and Withaferin A: Potential in Integrative Oncology. Int. J. Mol. Sci..

[15] Kashyap V.K., Dhasmana A., Yallapu M.M., Chauhan S.C., Jaggi M. (2020). *Withania somnifera* as a potential future drug molecule for COVID-19. Future Drug Discov..

[16] Siddiqui M.A., Farshori N.N., Al-Oqail M.M., Pant A.B., Al-Khedhairy A.A. (2021). Neuroprotective Effects of *Withania somnifera* on 4-Hydroxynonenal Induced Cell Death in Human Neuroblastoma SH-SY5Y Cells Through ROS Inhibition and Apoptotic Mitochondrial Pathway. Neurochem. Res..

[17] Peng S.Y., Wang Y.Y., Lan T.H., Lin L.C., Yuan S.F., Tang J.Y., Chang H.W. (2020). Low Dose Combined Treatment with Ultraviolet-C and Withaferin a Enhances Selective Killing of Oral Cancer Cells. Antioxidants.

[18] Heidari Z., Mahmoudzadeh-Sagheb H., Sarbishegi M., Gorgich E.A.C. (2021). Withania coagulans extract attenuates oxidative stress-mediated apoptosis of cerebellar purkinje neurons after ischemia/reperfusion injury. Metab. Brain Dis..

[19] Zhang X., Mukerji R., Samadi A.K., Cohen M.S. (2011). Down-regulation of estrogen receptor-alpha and rearranged during transfection tyrosine kinase is associated with withaferin a-induced apoptosis in MCF-7 breast cancer cells. BMC Complement. Altern. Med..

[20] Stan S.D., Hahm E.-R., Warin R., Singh S.V. (2008). Withaferin A causes FOXO3a-and Bim-dependent apoptosis and inhibits growth of human breast cancer cells in vivo. Cancer Res..

[21] Yang E.S., Choi M.J., Kim J.H., Choi K.S., Kwon T.K. (2011). Withaferin A enhances radiation-induced apoptosis in Caki cells through induction of reactive oxygen species, Bcl-2 downregulation and Akt inhibition. Chem.-Biol. Interact..

[22] Sinha P., Ostrand-Rosenberg S. (2013). Myeloid-derived suppressor cell function is reduced by Withaferin A, a potent and abundant component of *Withania somnifera* root extract. Cancer Immunol. Immunother..

[23] Kim S.H., Singh S.V. (2014). Mammary cancer chemoprevention by withaferin A is accompanied by in vivo suppression of self-renewal of cancer stem cells. Cancer Prev. Res..

[24] Chandrasekaran B., Pal D., Kolluru V., Tyagi A., Baby B., Dahiya N.R., Youssef K., Alatassi H., Ankem M.K., Sharma A.K. (2018). The chemopreventive effect of withaferin A on spontaneous and inflammation-associated colon carcinogenesis models. Carcinogenesis.

[25] Oh J.H., Kwon T.K. (2009). Withaferin A inhibits tumor necrosis factor-α-induced expression of cell adhesion molecules by inactivation of Akt and NF-κB in human pulmonary epithelial cells. Int. Immunopharmacol..

[26] Garg S., Huifu H., Kumari A., Sundar D., Kaul S.C., Wadhwa R. (2019). Induction of Senescence in Cancer Cells by a Novel Combination of Cucurbitacin B and Withanone: Molecular Mechanism and Therapeutic Potential. J. Gerontol. Ser. A.

[27] Dubey S., Yoon H., Cohen M.S., Nagarkatti P., Nagarkatti M., Karan D. (2018). Withaferin A Associated Differential Regulation of Inflammatory Cytokines. Front. Immunol..

[28] Tewari D., Chander V., Dhyani A., Sahu S., Gupta P., Patni P., Kalick L.S., Bishayee A. (2022). *Withania somnifera* (L.) Dunal: Phytochemistry, structure-activity relationship, and anticancer potential. Phytomedicine.

[29] Choudhary D., Bhattacharyya S., Bose S. (2017). Efficacy and Safety of Ashwagandha (*Withania somnifera* (L.) Dunal) Root Extract in Improving Memory and Cognitive Functions. J. Diet Suppl..

[30] Choudhary D., Bhattacharyya S., Joshi K. (2017). Body Weight Management in Adults Under Chronic Stress Through Treatment With Ashwagandha Root Extract: A Double-Blind, Randomized, Placebo-Controlled Trial. J. Evid. Based Complement. Altern. Med..

[31] Salve J., Pate S., Debnath K., Langade D. (2019). Adaptogenic and Anxiolytic Effects of Ashwagandha Root Extract in Healthy Adults: A Double-blind, Randomized, Placebo-controlled Clinical Study. Cureus.

[32] Chandrasekhar K., Kapoor J., Anishetty S. (2012). A prospective, randomized double-blind, placebo-controlled study of safety and efficacy of a high-concentration full-spectrum extract of ashwagandha root in reducing stress and anxiety in adults. Indian J. Psychol. Med..

[33] Kulkarni S.K., Dhir A. (2008). *Withania somnifera*: An Indian ginseng. Prog. Neuropsychopharmacol. Biol. Psychiatry.

[34] Dar N.J., Hamid A., Ahmad M. (2015). Pharmacologic overview of *Withania somnifera*, the Indian Ginseng. Cell Mol. Life Sci..

[35] Gorelick J., Rosenberg R., Smotrich A., Hanuš L., Bernstein N. (2015). Hypoglycemic activity of withanolides and elicitated *Withania somnifera*. Phytochemistry.

[36] Gupta A., Singh S. (2014). Evaluation of anti-inflammatory effect of *Withania somnifera* root on collagen-induced arthritis in rats. Pharm. Biol..

[37] Mohanty I.R., Arya D.S., Gupta S.K. (2008). *Withania somnifera* provides cardioprotection and attenuates ischemia-reperfusion induced apoptosis. Clin. Nutr..

[38] RajaSankar S., Manivasagam T., Sankar V., Prakash S., Muthusamy R., Krishnamurti A., Surendran S. (2009). *Withania somnifera* root extract improves catecholamines and physiological abnormalities seen in a Parkinson’s disease model mouse. J. Ethnopharmacol..

[39] Bhatnagar M., Goel I., Roy T., Shukla S.D., Khurana S. (2017). Complete Comparison Display (CCD) evaluation of ethanol extracts of Centella asiatica and *Withania somnifera* shows that they can non-synergistically ameliorate biochemical and behavioural damages in MPTP induced Parkinson’s model of mice. PLoS ONE.

[40] Chandrasekaran S., Dayakar A., Veronica J., Sundar S., Maurya R. (2013). An in vitro study of apoptotic like death in Leishmania donovani promastigotes by withanolides. Parasitol. Int..

[41] Sachdeva H., Sehgal R., Kaur S. (2013). Studies on the protective and immunomodulatory efficacy of *Withania somnifera* along with cisplatin against experimental visceral leishmaniasis. Parasitol. Res..

[42] Reuland D.J., Khademi S., Castle C.J., Irwin D.C., McCord J.M., Miller B.F., Hamilton K.L. (2013). Upregulation of phase II enzymes through phytochemical activation of Nrf2 protects cardiomyocytes against oxidant stress. Free Radic. Biol. Med..

[43] Budhiraja R.D., Sudhir S. (1987). Review of biological activity of withanolides. J. Sci. Ind. Res..

[44] Rastogi RP M.B. (1998). Compendium of Indian Medicinal Plants.

[45] Bone K. (1996). Clinical applications of Ayurvedic and Chinese herbs. Monograph for the Western Herbal Practitioner.

[46] Elsakka M., Grigorescu E., Stănescu U., Stănescu U., Dorneanu V. (1990). New data referring to chemistry of *Withania somnifera* species. Rev. Med. Chir. Soc. Med. Nat. Iasi.

[47] Srivastava D.N., Deshpande S.M. (1975). Gas chromatographic identification of fatty acids, fatty alcohols, and hydrocarbons of Convolvulus pluricaulis (Chois). J. Am. Oil Chem. Soc..

[48] Vedi M., Sabina E.P. (2016). Assessment of hepatoprotective and nephroprotective potential of withaferin A on bromobenzene-induced injury in Swiss albino mice: Possible involvement of mitochondrial dysfunction and inflammation. Cell Biol. Toxicol..

[49] Yan Z., Guo R., Gan L., Lau W.B., Cao X., Zhao J., Ma X., Christopher T.A., Lopez B.L., Wang Y. (2018). Withaferin A inhibits apoptosis via activated Akt-mediated inhibition of oxidative stress. Life Sci..

[50] Tiruveedi V.L., Bale S., Khurana A., Godugu C. (2018). Withaferin A, a novel compound of Indian ginseng (*Withania somnifera*), ameliorates Cerulein-induced acute pancreatitis: Possible role of oxidative stress and inflammation. Phytother. Res..

[51] Palliyaguru D.L., Chartoumpekis D.V., Wakabayashi N., Skoko J.J., Yagishita Y., Singh S.V., Kensler T.W. (2016). Withaferin A induces Nrf2-dependent protection against liver injury: Role of Keap1-independent mechanisms. Free Radic. Biol. Med..

[52] Bale S., Pulivendala G., Godugu C. (2018). Withaferin A attenuates bleomycin-induced scleroderma by targeting FoxO3a and NF-κβ signaling: Connecting fibrosis and inflammation. Biofactors.

[53] Anwar M.F., Yadav D., Rastogi S., Arora I., Khar R.K., Chander J., Samim M. (2015). Modulation of liver and kidney toxicity by herb *Withania somnifera* for silver nanoparticles: A novel approach for harmonizing between safety and use of nanoparticles. Protoplasma.

[54] Biswal B.M., Sulaiman S.A., Ismail H.C., Zakaria H., Musa K.I. (2013). Effect of *Withania somnifera* (Ashwagandha) on the development of chemotherapy-induced fatigue and quality of life in breast cancer patients. Integr. Cancer Ther..

[55] Zhang X., Samadi A.K., Roby K.F., Timmermann B., Cohen M.S. (2012). Inhibition of cell growth and induction of apoptosis in ovarian carcinoma cell lines CaOV3 and SKOV3 by natural withanolide Withaferin A. Gynecol. Oncol..

[56] Thaiparambil J.T., Bender L., Ganesh T., Kline E., Patel P., Liu Y., Tighiouart M., Vertino P.M., Harvey R.D., Garcia A. (2011). Withaferin A inhibits breast cancer invasion and metastasis at sub-cytotoxic doses by inducing vimentin disassembly and serine 56 phosphorylation. Int. J. Cancer.

[57] Oh J.H., Lee T.-J., Kim S.H., Choi Y.H., Lee S.H., Lee J.M., Kim Y.-H., Park J.-W., Kwon T.K. (2008). Induction of apoptosis by withaferin A in human leukemia U937 cells through down-regulation of Akt phosphorylation. Apoptosis.

[58] Widodo N., Kaur K., Shrestha B.G., Takagi Y., Ishii T., Wadhwa R., Kaul S.C. (2007). Selective killing of cancer cells by leaf extract of Ashwagandha: Identification of a tumor-inhibitory factor and the first molecular insights to its effect. Clin. Cancer Res..

[59] Widodo N., Takagi Y., Shrestha B.G., Ishii T., Kaul S.C., Wadhwa R. (2008). Selective killing of cancer cells by leaf extract of Ashwagandha: Components, activity and pathway analyses. Cancer Lett..

[60] Yang H., Shi G., Dou Q.P. (2007). The tumor proteasome is a primary target for the natural anticancer compound Withaferin A isolated from “Indian winter cherry”. Mol. Pharmacol..

[61] Samadi A.K., Mukerji R., Shah A., Timmermann B.N., Cohen M.S. (2010). A novel RET inhibitor with potent efficacy against medullary thyroid cancer in vivo. Surgery.

[62] Yang H., Wang Y., Cheryan V.T., Wu W., Cui C.Q., Polin L.A., Pass H.I., Dou Q.P., Rishi A.K., Wali A. (2012). Withaferin A inhibits the proteasome activity in mesothelioma in vitro and in vivo. PLoS ONE.

[63] Yu Y., Hamza A., Zhang T., Gu M., Zou P., Newman B., Li Y., Gunatilaka A.A., Zhan C.G., Sun D. (2010). Withaferin A targets heat shock protein 90 in pancreatic cancer cells. Biochem. Pharmacol..

[64] Manoharan S., Panjamurthy K., Menon V.P., Balakrishnan S., Alias L.M. (2009). Protective effect of Withaferin-A on tumour formation in 7,12-dimethylbenz[a]anthracene induced oral carcinogenesis in hamsters. Indian J. Exp. Biol..

[65] Hahm E.R., Lee J., Singh S.V. (2014). Role of mitogen-activated protein kinases and Mcl-1 in apoptosis induction by withaferin A in human breast cancer cells. Mol. Carcinog..

[66] Devi P.U., Sharada A.C., Solomon F.E., Kamath M.S. (1992). In vivo growth inhibitory effect of *Withania somnifera* (Ashwagandha) on a transplantable mouse tumor, Sarcoma 180. Indian J. Exp. Biol..

[67] Munagala R., Kausar H., Munjal C., Gupta R.C. (2011). Withaferin A induces p53-dependent apoptosis by repression of HPV oncogenes and upregulation of tumor suppressor proteins in human cervical cancer cells. Carcinogenesis.

[68] Xia S., Miao Y., Liu S. (2018). Withaferin A induces apoptosis by ROS-dependent mitochondrial dysfunction in human colorectal cancer cells. Biochem. Biophys. Res. Commun..

[69] Chang E., Pohling C., Natarajan A., Witney T.H., Kaur J., Xu L., Gowrishankar G., D’Souza A.L., Murty S., Schick S. (2016). AshwaMAX and Withaferin A inhibits gliomas in cellular and murine orthotopic models. J. Neurooncol..

[70] Li X., Zhu F., Jiang J., Sun C., Zhong Q., Shen M., Wang X., Tian R., Shi C., Xu M. (2016). Simultaneous inhibition of the ubiquitin-proteasome system and autophagy enhances apoptosis induced by ER stress aggravators in human pancreatic cancer cells. Autophagy.

[71] Mandal C., Dutta A., Mallick A., Chandra S., Misra L., Sangwan R.S., Mandal C. (2008). Withaferin A induces apoptosis by activating p38 mitogen-activated protein kinase signaling cascade in leukemic cells of lymphoid and myeloid origin through mitochondrial death cascade. Apoptosis.

[72] Malik F., Kumar A., Bhushan S., Khan S., Bhatia A., Suri K.A., Qazi G.N., Singh J. (2007). Reactive oxygen species generation and mitochondrial dysfunction in the apoptotic cell death of human myeloid leukemia HL-60 cells by a dietary compound withaferin A with concomitant protection by N-acetyl cysteine. Apoptosis.

[73] Srinivasan S., Ranga R.S., Burikhanov R., Han S.S., Chendil D. (2007). Par-4-dependent apoptosis by the dietary compound withaferin A in prostate cancer cells. Cancer Res..

[74] Abutaha N. (2015). In vitro antiproliferative activity of partially purified *Withania somnifera* fruit extract on different cancer cell lines. J. Balkan Union Oncol..

[75] Mohan R., Hammers H.J., Bargagna-Mohan P., Zhan X.H., Herbstritt C.J., Ruiz A., Zhang L., Hanson A.D., Conner B.P., Rougas J. (2004). Withaferin A is a potent inhibitor of angiogenesis. Angiogenesis.

[76] Ahmed W., Mofed D., Zekri A.-R., El-Sayed N., Rahouma M., Sabet S. (2018). Antioxidant activity and apoptotic induction as mechanisms of action of *Withania somnifera* (Ashwagandha) against a hepatocellular carcinoma cell line. J. Int. Med. Res..

[77] Yu Y., Katiyar S.P., Sundar D., Kaul Z., Miyako E., Zhang Z., Kaul S.C., Reddel R.R., Wadhwa R. (2017). Withaferin-A kills cancer cells with and without telomerase: Chemical, computational and experimental evidences. Cell Death Dis..

[78] Mayola E., Gallerne C., Esposti D.D., Martel C., Pervaiz S., Larue L., Debuire B., Lemoine A., Brenner C., Lemaire C. (2011). Withaferin A induces apoptosis in human melanoma cells through generation of reactive oxygen species and down-regulation of Bcl-2. Apoptosis.

[79] Hsu J.H., Chang P.M., Cheng T.S., Kuo Y.L., Wu A.T., Tran T.H., Yang Y.H., Chen J.M., Tsai Y.C., Chu Y.S. (2019). Identification of Withaferin A as a Potential Candidate for Anti-Cancer Therapy in Non-Small Cell Lung Cancer. Cancers.

[80] McKenna M.K., Gachuki B.W., Alhakeem S.S., Oben K.N., Rangnekar V.M., Gupta R.C., Bondada S. (2015). Anti-cancer activity of withaferin A in B-cell lymphoma. Cancer Biol. Ther..

[81] Nishikawa Y., Okuzaki D., Fukushima K., Mukai S., Ohno S., Ozaki Y., Yabuta N., Nojima H. (2015). Withaferin A Induces Cell Death Selectively in Androgen-Independent Prostate Cancer Cells but Not in Normal Fibroblast Cells. PLoS ONE.

[82] Das T., Roy K.S., Chakrabarti T., Mukhopadhyay S., Roychoudhury S. (2014). Withaferin A modulates the Spindle assembly checkpoint by degradation of Mad2-Cdc20 complex in colorectal cancer cell lines. Biochem. Pharmacol..

[83] Vaishnavi K., Saxena N., Shah N., Singh R., Manjunath K., Uthayakumar M., Kanaujia S.P., Kaul S.C., Sekar K., Wadhwa R. (2012). Differential Activities of the Two Closely Related Withanolides, Withaferin A and Withanone: Bioinformatics and Experimental Evidences. PLoS ONE.

[84] Sari A.N., Bhargava P., Dhanjal J.K., Putri J.F., Radhakrishnan N., Shefrin S., Ishida Y., Terao K., Sundar D., Kaul S.C. (2020). Combination of Withaferin-A and CAPE Provides Superior Anticancer Potency: Bioinformatics and Experimental Evidence to Their Molecular Targets and Mechanism of Action. Cancers.

[85] Sundar D., Yu Y., Katiyar S.P., Putri J.F., Dhanjal J.K., Wang J., Sari A.N., Kolettas E., Kaul S.C., Wadhwa R. (2019). Wild type p53 function in p53Y220C mutant harboring cells by treatment with Ashwagandha derived anticancer withanolides: Bioinformatics and experimental evidence. J. Exp. Clin. Cancer Res..

[86] Choudhary M.I., Hussain S., Yousuf S., Dar A., Mudassar, Attaur R. (2010). Chlorinated and diepoxy withanolides from *Withania somnifera* and their cytotoxic effects against human lung cancer cell line. Phytochemistry.

[87] Oza V.P., Parmar P.P., Kumar S., Subramanian R.B. (2010). Anticancer properties of highly purified L-asparaginase from *Withania somnifera* L. against acute lymphoblastic leukemia. Appl. Biochem. Biotechnol..

[88] Leyon P.V., Kuttan G. (2004). Effect of *Withania somnifera* on B16F-10 melanoma induced metastasis in mice. Phytother. Res..

[89] Bani S., Gautam M., Sheikh F.A., Khan B., Satti N.K., Suri K.A., Qazi G.N., Patwardhan B. (2006). Selective Th1 up-regulating activity of *Withania somnifera* aqueous extract in an experimental system using flow cytometry. J. Ethnopharmacol..

[90] Zhao J., Nakamura N., Hattori M., Kuboyama T., Tohda C., Komatsu K. (2002). Withanolide derivatives from the roots of *Withania somnifera* and their neurite outgrowth activities. Chem. Pharm. Bull..

[91] Wadhwa R., Singh R., Gao R., Shah N., Widodo N., Nakamoto T., Ishida Y., Terao K., Kaul S.C. (2013). Water Extract of Ashwagandha Leaves Has Anticancer Activity: Identification of an Active Component and Its Mechanism of Action. PLoS ONE.

[92] Jayaprakasam B., Zhang Y., Seeram N.P., Nair M.G. (2003). Growth inhibition of human tumor cell lines by withanolides from *Withania somnifera* leaves. Life Sci..

[93] Rah B., Amin H., Yousuf K., Khan S., Jamwal G., Mukherjee D., Goswami A. (2012). A novel MMP-2 inhibitor 3-azidowithaferin A (3-azidoWA) abrogates cancer cell invasion and angiogenesis by modulating extracellular Par-4. PLoS ONE.

[94] Wang H.-C., Tsai Y.-L., Wu Y.-C., Chang F.-R., Liu M.-H., Chen W.-Y., Wu C.-C. (2012). Withanolides-Induced Breast Cancer Cell Death Is Correlated with Their Ability to Inhibit Heat Protein 90. PLoS ONE.

[95] Wang H.-C., Hu H.-H., Chang F.-R., Tsai J.-Y., Kuo C.-Y., Wu Y.-C., Wu C.-C. (2019). Different effects of 4β-hydroxywithanolide E and withaferin A, two withanolides from Solanaceae plants, on the Akt signaling pathway in human breast cancer cells. Phytomedicine.

[96] Yadav D.K., Kumar S., Saloni, Singh H., Kim M.H., Sharma P., Misra S., Khan F. (2017). Molecular docking, QSAR and ADMET studies of withanolide analogs against breast cancer. Drug Des. Dev. Ther..

[97] Mondal S., Mandal C., Sangwan R., Chandra S., Mandal C. (2010). Withanolide D induces apoptosis in leukemia by targeting the activation of neutral sphingomyelinase-ceramide cascade mediated by synergistic activation of c-Jun N-terminal kinase and p38 mitogen-activated protein kinase. Mol. Cancer.

[98] Issa M.E., Wijeratne E.M.K., Gunatilaka A.A.L., Cuendet M. (2017). Withanolide D Exhibits Similar Cytostatic Effect in Drug-Resistant and Drug-Sensitive Multiple Myeloma Cells. Front. Pharmacol..

[99] Kataria H., Shah N., Kaul S.C., Wadhwa R., Kaur G. (2011). Water extract of ashwagandha leaves limits proliferation and migration, and induces differentiation in glioma cells. Evid.-Based Complement. Altern..

[100] Widodo N., Priyandoko D., Shah N., Wadhwa R., Kaul S.C. (2010). Selective Killing of Cancer Cells by Ashwagandha Leaf Extract and Its Component Withanone Involves ROS Signaling. PLoS ONE.

[101] Kim S.-H., Singh K.B., Hahm E.-R., Lokeshwar B.L., Singh S.V. (2020). *Withania somnifera* root extract inhibits fatty acid synthesis in prostate cancer cells. J. Tradit. Complement. Med..

[102] Setty Balakrishnan A., Nathan A.A., Kumar M., Ramamoorthy S., Ramia Mothilal S.K. (2017). *Withania somnifera* targets interleukin-8 and cyclooxygenase-2 in human prostate cancer progression. Prostate Int..

[103] Henley A.B., Yang L., Chuang K.-L., Sahuri-Arisoylu M., Wu L.-H., Bligh S.W.A., Bell J.D. (2017). *Withania somnifera* Root Extract Enhances Chemotherapy through ‘Priming’. PLoS ONE.

[104] Kunnumakkara A.B., Harsha C., Banik K., Vikkurthi R., Sailo B.L., Bordoloi D., Gupta S.C., Aggarwal B.B. (2019). Is curcumin bioavailability a problem in humans: Lessons from clinical trials. Expert. Opin. Drug Metab. Toxicol..

[105] Dai T., Jiang W., Guo Z., Wang Z., Huang M., Zhong G., Liang C., Pei X., Dai R. (2019). Studies on oral bioavailability and first-pass metabolism of withaferin A in rats using LC-MS/MS and Q-TRAP. Biomed. Chromatogr..

[106] Patil D., Gautam M., Mishra S., Karupothula S., Gairola S., Jadhav S., Pawar S., Patwardhan B. (2013). Determination of withaferin A and withanolide A in mice plasma using high-performance liquid chromatography-tandem mass spectrometry: Application to pharmacokinetics after oral administration of *Withania somnifera* aqueous extract. J. Pharm. Biomed. Anal..

[107] Senthilnathan P., Padmavathi R., Banu S.M., Sakthisekaran D. (2006). Enhancement of antitumor effect of paclitaxel in combination with immunomodulatory *Withania somnifera* on benzo (a) pyrene induced experimental lung cancer. Chem.-Biol. Interact..

[108] Senthilnathan P., Padmavathi R., Magesh V., Sakthisekaran D. (2006). Chemotherapeutic efficacy of paclitaxel in combination with *Withania somnifera* on benzo (a) pyrene-induced experimental lung cancer. Cancer Sci..

[109] Cai Y., Sheng Z.-Y., Chen Y., Bai C. (2014). Effect of Withaferin A on A549 cellular proliferation and apoptosis in non-small cell lung cancer. Asian Pac. J. Cancer Prev..

[110] Kyakulaga A.H., Aqil F., Munagala R., Gupta R.C. (2018). Withaferin A inhibits Epithelial to Mesenchymal Transition in Non-Small Cell Lung Cancer Cells. Sci. Rep..

[111] Bray F., Ferlay J., Soerjomataram I., Siegel R.L., Torre L.A., Jemal A. (2018). Global cancer statistics 2018: GLOBOCAN estimates of incidence and mortality worldwide for 36 cancers in 185 countries. CA Cancer J. Clin..

[112] Arpino G., Milano M., De Placido S. (2015). Features of aggressive breast cancer. Breast.

[113] Stan S.D., Zeng Y., Singh S.V. (2008). Ayurvedic medicine constituent withaferin a causes G2 and M phase cell cycle arrest in human breast cancer cells. Nutr. Cancer.

[114] Hahm E.-R., Singh S.V. (2013). Withaferin A-induced apoptosis in human breast cancer cells is associated with suppression of inhibitor of apoptosis family protein expression. Cancer Lett..

[115] Hahm E.-R., Moura M.B., Kelley E.E., Van Houten B., Shiva S., Singh S.V. (2011). Withaferin A-induced apoptosis in human breast cancer cells is mediated by reactive oxygen species. PLoS ONE.

[116] Bargagna-Mohan P., Hamza A., Kim Y.-e., Ho Y.K.A., Mor-Vaknin N., Wendschlag N., Liu J., Evans R.M., Markovitz D.M., Zhan C.-G. (2007). The tumor inhibitor and antiangiogenic agent withaferin A targets the intermediate filament protein vimentin. Chem. Biol..

[117] Yang Z., Garcia A., Xu S., Powell D.R., Vertino P.M., Singh S., Marcus A.I. (2013). *Withania somnifera* root extract inhibits mammary cancer metastasis and epithelial to mesenchymal transition. PLoS ONE.

[118] Khazal K.F., Hill D.L. (2015). *Withania somnifera* extract reduces the invasiveness of MDA-MB-231 breast cancer and inhibits cytokines associated with metastasis. J. Cancer Metastasis Treat..

[119] Khazal K.F., Samuel T., Hill D.L., Grubbs C.J. (2013). Effect of an extract of *Withania somnifera* root on estrogen receptor-positive mammary carcinomas. Anticancer Res..

[120] Hahm E.R., Lee J., Huang Y., Singh S.V. (2011). Withaferin a suppresses estrogen receptor-α expression in human breast cancer cells. Mol. Carcinog..

[121] Lee J., Hahm E.R., Marcus A.I., Singh S.V. (2015). Withaferin A inhibits experimental epithelial-mesenchymal transition in MCF-10A cells and suppresses vimentin protein level in vivo in breast tumors. Mol. Carcinog..

[122] Lee J., Hahm E.-R., Singh S.V. (2010). Withaferin A inhibits activation of signal transducer and activator of transcription 3 in human breast cancer cells. Carcinogenesis.

[123] Mulabagal V., Subbaraju G.V., Rao C.V., Sivaramakrishna C., DeWitt D.L., Holmes D., Sung B., Aggarwal B.B., Tsay H.S., Nair M.G. (2009). Withanolide sulfoxide from Aswagandha roots inhibits nuclear transcription factor-kappa-B, cyclooxygenase and tumor cell proliferation. Phytother. Res. Int. J. Devoted Pharmacol. Toxicol. Eval. Nat. Prod. Deriv..

[124] Lee J., Sehrawat A., Singh S.V. (2012). Withaferin A causes activation of Notch2 and Notch4 in human breast cancer cells. Breast Cancer Res. Treat..

[125] Nagalingam A., Kuppusamy P., Singh S.V., Sharma D., Saxena N.K. (2014). Mechanistic elucidation of the antitumor properties of withaferin a in breast cancer. Cancer Res..

[126] Vel Szic K.S., Declerck K., Crans R.A., Diddens J., Scherf D.B., Gerhäuser C., Berghe W.V. (2017). Epigenetic silencing of triple negative breast cancer hallmarks by Withaferin A. Oncotarget.

[127] Roy R.V., Suman S., Das T.P., Luevano J.E., Damodaran C. (2013). Withaferin A, a steroidal lactone from *Withania somnifera*, induces mitotic catastrophe and growth arrest in prostate cancer cells. J. Nat. Prod..

[128] Kunimasa K., Nagano T., Shimono Y., Dokuni R., Kiriu T., Tokunaga S., Tamura D., Yamamoto M., Tachihara M., Kobayashi K. (2017). Glucose metabolism-targeted therapy and withaferin A are effective for epidermal growth factor receptor tyrosine kinase inhibitor-induced drug-tolerant persisters. Cancer Sci..

[129] Muralikrishnan G., Dinda A.K., Shakeel F. (2010). Immunomodulatory effects of *Withania somnifera* on azoxymethane induced experimental colon cancer in mice. Immunol. Investig..

[130] Koduru S., Kumar R., Srinivasan S., Evers M.B., Damodaran C. (2010). Notch-1 inhibition by Withaferin-A: A therapeutic target against colon carcinogenesis. Mol. Cancer Ther..

[131] Choi B.Y., Kim B.-W. (2015). Withaferin-A inhibits colon cancer cell growth by blocking STAT3 transcriptional activity. J. Cancer Prev..

[132] Alnuqaydan A.M., Rah B., Almutary A.G., Chauhan S.S. (2020). Synergistic antitumor effect of 5-fluorouracil and withaferin-A induces endoplasmic reticulum stress-mediated autophagy and apoptosis in colorectal cancer cells. Am. J. Cancer Res..

[133] Yang E.S., Choi M.J., Kim J.H., Choi K.S., Kwon T.K. (2011). Combination of withaferin A and X-ray irradiation enhances apoptosis in U937 cells. Toxicol. In Vitro.

[134] Okamoto S., Tsujioka T., Suemori S., Kida J., Kondo T., Tohyama Y., Tohyama K. (2016). Withaferin A suppresses the growth of myelodysplasia and leukemia cell lines by inhibiting cell cycle progression. Cancer Sci..

[135] Turrini E., Calcabrini C., Sestili P., Catanzaro E., de Gianni E., Diaz A.R., Hrelia P., Tacchini M., Guerrini A., Canonico B. (2016). *Withania somnifera* Induces Cytotoxic and Cytostatic Effects on Human T Leukemia Cells. Toxins.

[136] Prakash J., Gupta S.K., Dinda A.K. (2002). *Withania somnifera* root extract prevents DMBA-induced squamous cell carcinoma of skin in Swiss albino mice. Nutr. Cancer..

[137] Chang H.-W., Li R.-N., Wang H.-R., Liu J.-R., Tang J.-Y., Huang H.-W., Chan Y.-H., Yen C.-Y. (2017). Withaferin A induces oxidative stress-mediated apoptosis and DNA damage in oral cancer cells. Front. Physiol..

[138] Lv T.Z., Wang G.S. (2015). Antiproliferation potential of withaferin A on human osteosarcoma cells via the inhibition of G2/M checkpoint proteins. Exp. Ther. Med..

[139] Kim G., Kim T.H., Hwang E.H., Chang K.T., Hong J.J., Park J.H. (2017). Withaferin A inhibits the proliferation of gastric cancer cells by inducing G2/M cell cycle arrest and apoptosis. Oncol. Lett..

[140] Li X., Zhu F., Jiang J., Sun C., Wang X., Shen M., Tian R., Shi C., Xu M., Peng F. (2015). Synergistic antitumor activity of withaferin A combined with oxaliplatin triggers reactive oxygen species-mediated inactivation of the PI3K/AKT pathway in human pancreatic cancer cells. Cancer lett..

[141] Fong M.Y., Jin S., Rane M., Singh R.K., Gupta R., Kakar S.S. (2012). Withaferin A synergizes the therapeutic effect of doxorubicin through ROS-mediated autophagy in ovarian cancer. PLoS ONE.

[142] Kakar S.S., Ratajczak M.Z., Powell K.S., Moghadamfalahi M., Miller D.M., Batra S.K., Singh S.K. (2014). Withaferin a alone and in combination with cisplatin suppresses growth and metastasis of ovarian cancer by targeting putative cancer stem cells. PLoS ONE.

[143] Lee D.H., Lim I.-H., Sung E.-G., Kim J.-Y., Song I.-H., Park Y.K., Lee T.-J. (2013). Withaferin A inhibits matrix metalloproteinase-9 activity by suppressing the Akt signaling pathway. Oncol. Rep..

[144] Um H.J., Min K.-j., Kim D.E., Kwon T.K. (2012). Withaferin A inhibits JAK/STAT3 signaling and induces apoptosis of human renal carcinoma Caki cells. Biochem. Biophys. Res. Commun..

[145] Choi M.J., Park E.J., Min K.J., Park J.W., Kwon T.K. (2011). Endoplasmic reticulum stress mediates withaferin A-induced apoptosis in human renal carcinoma cells. Toxicol In Vitro.

[146] Heyninck K., Lahtela-Kakkonen M., Van der Veken P., Haegeman G., Berghe W.V. (2014). Withaferin A inhibits NF-kappaB activation by targeting cysteine 179 in IKKβ. Biochem. Pharmacol..

[147] Tripathi S.K., Pandey K., Panda M., Spinella M.J., Rengasamy K.R.R., Biswal B.K. (2019). The potential of retinoids for combination therapy of lung cancer: Updates and future directions. Pharmacol. Res..

[148] Kyakulaga A.H., Aqil F., Munagala R., Gupta R.C. (2020). Synergistic combinations of paclitaxel and withaferin A against human non-small cell lung cancer cells. Oncotarget.

[149] Suttana W., Mankhetkorn S., Poompimon W., Palagani A., Zhokhov S., Gerlo S., Haegeman G., Berghe W.V. (2010). Differential chemosensitization of P-glycoprotein overexpressing K562/Adr cells by withaferin A and Siamois polyphenols. Mol. Cancer.

[150] Rah B., ur Rasool R., Nayak D., Yousuf S.K., Mukherjee D., Kumar L.D., Goswami A. (2015). PAWR-mediated suppression of BCL2 promotes switching of 3-azido withaferin A (3-AWA)-induced autophagy to apoptosis in prostate cancer cells. Autophagy.

[151] Grogan P.T., Sleder K.D., Samadi A.K., Zhang H., Timmermann B.N., Cohen M.S. (2013). Cytotoxicity of withaferin A in glioblastomas involves induction of an oxidative stress-mediated heat shock response while altering Akt/mTOR and MAPK signaling pathways. Investig. New Drugs.

[152] Grogan P.T., Sarkaria J.N., Timmermann B.N., Cohen M.S. (2014). Oxidative cytotoxic agent withaferin A resensitizes temozolomide-resistant glioblastomas via MGMT depletion and induces apoptosis through Akt/mTOR pathway inhibitory modulation. Investig. New Drugs..

[153] Sun G.Y., Li R., Cui J., Hannink M., Gu Z., Fritsche K.L., Lubahn D.B., Simonyi A. (2016). *Withania somnifera* and Its Withanolides Attenuate Oxidative and Inflammatory Responses and Up-Regulate Antioxidant Responses in BV-2 Microglial Cells. Neuromolecular Med..

[154] Kataria H., Wadhwa R., Kaul S.C., Kaur G. (2013). *Withania somnifera* water extract as a potential candidate for differentiation based therapy of human neuroblastomas. PLoS ONE.

[155] Kataria H., Kumar S., Chaudhary H., Kaur G. (2016). *Withania somnifera* Suppresses Tumor Growth of Intracranial Allograft of Glioma Cells. Mol. Neurobiol..

[156] Ting L.-L., Chou A.S.-B., Hsieh C.-H., Hsiung S.-C., Pang S.-T., Liao S.-K. (2016). Withaferin A targeting both cancer stem cells and metastatic cancer stem cells in the UP-LN1 carcinoma cell model. J. Cancer Metastatis Treat..

[157] Cohen S.M., Mukerji R., Timmermann B.N., Samadi A.K., Cohen M.S. (2012). A novel combination of withaferin A and sorafenib shows synergistic efficacy against both papillary and anaplastic thyroid cancers. Am. J. Surg..

[158] Pilot Study of Curcumin Formulation and Ashwagandha Extract in Advanced Osteosarcoma (OSCAT). https://clinicaltrials.gov/ct2/show/results/NCT00689195?term=Withania+somnifera&cond=cancer&draw=2&rank=3.

[159] Bhat J., Damle A., Vaishnav P.P., Albers R., Joshi M., Banerjee G. (2010). In vivo enhancement of natural killer cell activity through tea fortified with Ayurvedic herbs. Phytother. Res..

[160] Ashwagandha: Effects on Stress, Inflammation and Immune Cell Activation. https://clinicaltrials.gov/ct2/show/NCT00817752?term=Withania+somnifera&cond=cancer&draw=2&rank=2.

[161] Chengappa K.N.R., Brar J.S., Gannon J.M., Schlicht P.J. (2018). Adjunctive Use of a Standardized Extract of *Withania somnifera* (Ashwagandha) to Treat Symptom Exacerbation in Schizophrenia: A Randomized, Double-Blind, Placebo-Controlled Study. J. Clin. Psychiatry.

[162] Mandlik Ingawale D.S., Namdeo A.G. (2021). Pharmacological evaluation of Ashwagandha highlighting its healthcare claims, safety, and toxicity aspects. J. Diet Suppl..

[163] Khanal P., Chikhale R., Dey Y.N., Pasha I., Chand S., Gurav N., Ayyanar M., Patil B.M., Gurav S. (2021). Withanolides from *Withania somnifera* as an immunity booster and their therapeutic options against COVID-19. J. Biomol. Struct. Dyn..

[164] Chandran U., Patwardhan B. (2017). Network ethnopharmacological evaluation of the immunomodulatory activity of *Withania somnifera*. J. Ethnopharmacol..

[165] Turner M.D., Nedjai B., Hurst T., Pennington D.J. (2014). Cytokines and chemokines: At the crossroads of cell signalling and inflammatory disease. Biochim. Biophys. Acta (BBA)-Mol. Cell Res..

[166] Liu T., Zhang L., Joo D., Sun S.-C. (2017). NF-κB signaling in inflammation. Signal Transduct. Target. Ther..

[167] Ghosh S., May M.J., Kopp E.B. (1998). NF-kappa B and Rel proteins: Evolutionarily conserved mediators of immune responses. Annu Rev. Immunol..

[168] Vanden Berghe W., Sabbe L., Kaileh M., Haegeman G., Heyninck K. (2012). Molecular insight in the multifunctional activities of Withaferin A. Biochem. Pharmacol..

[169] Iuvone T., Esposito G., Capasso F., Izzo A.A. (2003). Induction of nitric oxide synthase expression by *Withania somnifera* in macrophages. Life Sci..

[170] Min K.J., Choi K., Kwon T.K. (2011). Withaferin A down-regulates lipopolysaccharide-induced cyclooxygenase-2 expression and PGE2 production through the inhibition of STAT1/3 activation in microglial cells. Int. Immunopharmacol..

[171] Singh D., Aggarwal A., Maurya R., Naik S. (2007). *Withania somnifera* inhibits NF-kappaB and AP-1 transcription factors in human peripheral blood and synovial fluid mononuclear cells. Phytother. Res..

[172] Russo A., Izzo A.A., Cardile V., Borrelli F., Vanella A. (2001). Indian medicinal plants as antiradicals and DNA cleavage protectors. Phytomedicine.

[173] Davis L., Kuttan G. (2000). Immunomodulatory activity of *Withania somnifera*. J. Ethnopharmacol..

[174] Ziauddin M., Phansalkar N., Patki P., Diwanay S., Patwardhan B. (1996). Studies on the immunomodulatory effects of Ashwagandha. J. Ethnopharmacol..

[175] Oh J.H., Lee T.J., Park J.W., Kwon T.K. (2008). Withaferin A inhibits iNOS expression and nitric oxide production by Akt inactivation and down-regulating LPS-induced activity of NF-kappaB in RAW 264.7 cells. Eur. J. Pharmacol..

[176] Chauhan D.S., Dhasmana A., Laskar P., Prasad R., Jain N.K., Srivastava R., Jaggi M., Chauhan S.C., Yallapu M.M. (2021). Nanotechnology synergized immunoengineering for cancer. Eur. J. Pharm. Biopharm..

[177] Shetty A., Nagesh P.K.B., Setua S., Hafeez B.B., Jaggi M., Yallapu M.M., Chauhan S.C. (2020). Novel Paclitaxel Nanoformulation Impairs De Novo Lipid Synthesis in Pancreatic Cancer Cells and Enhances Gemcitabine Efficacy. ACS Omega.

[178] Samanta K., Setua S., Kumari S., Jaggi M., Yallapu M.M., Chauhan S.C. (2019). Gemcitabine Combination Nano Therapies for Pancreatic Cancer. Pharmaceutics.

[179] Massey A.E., Sikander M., Chauhan N., Kumari S., Setua S., Shetty A.B., Mandil H., Kashyap V.K., Khan S., Jaggi M. (2019). Next-generation paclitaxel-nanoparticle formulation for pancreatic cancer treatment. Nanomedicine.

[180] Kim B., Park J.E., Im E., Cho Y., Lee J., Lee H.J., Sim D.Y., Park W.Y., Shim B.S., Kim S.H. (2021). Recent Advances in Nanotechnology with Nano-Phytochemicals: Molecular Mechanisms and Clinical Implications in Cancer Progression. Int. J. Mol. Sci..

[181] Salama L., Pastor E.R., Stone T., Mousa S.A. (2020). Emerging Nanopharmaceuticals and Nanonutraceuticals in Cancer Management. Biomedicines.

[182] Granja A., Frias I., Neves A.R., Pinheiro M., Reis S. (2017). Therapeutic Potential of Epigallocatechin Gallate Nanodelivery Systems. BioMed Res. Int..

[183] Khan T., Gurav P. (2018). PhytoNanotechnology: Enhancing Delivery of Plant Based Anti-cancer Drugs. Front. Pharmacol..

[184] Wilhelm S., Tavares A.J., Dai Q., Ohta S., Audet J., Dvorak H.F., Chan W.C.W. (2016). Analysis of nanoparticle delivery to tumours. Nat. Rev. Mater..

[185] Khan S., Setua S., Kumari S., Dan N., Massey A., Hafeez B.B., Yallapu M.M., Stiles Z.E., Alabkaa A., Yue J. (2019). Superparamagnetic iron oxide nanoparticles of curcumin enhance gemcitabine therapeutic response in pancreatic cancer. Biomaterials.

[186] Nagesh P.K.B., Chowdhury P., Hatami E., Boya V.K.N., Kashyap V.K., Khan S., Hafeez B.B., Chauhan S.C., Jaggi M., Yallapu M.M. (2018). miRNA-205 Nanoformulation Sensitizes Prostate Cancer Cells to Chemotherapy. Cancers.

[187] Nanomedicine Market Is Estimated To Be Valued At $350.8 Billion By 2025: Grand View Research, Inc. https://www.grandviewresearch.com/press-release/global-nanomedicine-market.

[188] Anselmo A.C., Mitragotri S. (2015). A Review of Clinical Translation of Inorganic Nanoparticles. AAPS J..

[189] Zhang C., Yan L., Gu Z., Zhao Y. (2019). Strategies based on metal-based nanoparticles for hypoxic-tumor radiotherapy. Chem. Sci..

[190] Ju Y., Dong B., Yu J., Hou Y. (2019). Inherent multifunctional inorganic nanomaterials for imaging-guided cancer therapy. Nano Today.

[191] Hess K.L., Medintz I.L., Jewell C.M. (2019). Designing inorganic nanomaterials for vaccines and immunotherapies. Nano Today.

[192] Tian G., Zhang X., Gu Z., Zhao Y. (2015). Recent advances in upconversion nanoparticles-based multifunctional nanocomposites for combined cancer therapy. Adv. Mater..

[193] Kim T., Hyeon T. (2013). Applications of inorganic nanoparticles as therapeutic agents. Nanotechnology.

[194] Erathodiyil N., Ying J.Y. (2011). Functionalization of inorganic nanoparticles for bioimaging applications. Acc. Chem. Res..

[195] Na H.B., Song I.C., Hyeon T. (2009). Inorganic nanoparticles for MRI contrast agents. Adv. Mater..

[196] Liu J., Zheng X., Yan L., Zhou L., Tian G., Yin W., Wang L., Liu Y., Hu Z., Gu Z. (2015). Bismuth sulfide nanorods as a precision nanomedicine for in vivo multimodal imaging-guided photothermal therapy of tumor. ACS Nano.

[197] Tian G., Gu Z., Zhou L., Yin W., Liu X., Yan L., Jin S., Ren W., Xing G., Li S. (2012). Mn2+ dopant-controlled synthesis of NaYF4: Yb/Er upconversion nanoparticles for in vivo imaging and drug delivery. Adv. Mater..

[198] Du J., Gu Z., Yan L., Yong Y., Yi X., Zhang X., Liu J., Wu R., Ge C., Chen C. (2017). Poly(Vinylpyrollidone)- and Selenocysteine-Modified Bi(2) Se(3) Nanoparticles Enhance Radiotherapy Efficacy in Tumors and Promote Radioprotection in Normal Tissues. Adv. Mater..

[199] Samia A.C., Chen X., Burda C. (2003). Semiconductor quantum dots for photodynamic therapy. J. Am. Chem. Soc..

[200] Son S.J., Bai X., Lee S.B. (2007). Inorganic hollow nanoparticles and nanotubes in nanomedicine Part 1. Drug/gene delivery applications. Drug Discov. Today.

[201] Zhang C., Yan L., Wang X., Dong X., Zhou R., Gu Z., Zhao Y. (2019). Tumor Microenvironment-Responsive Cu(2)(OH)PO(4) Nanocrystals for Selective and Controllable Radiosentization via the X-ray-Triggered Fenton-like Reaction. Nano Lett..

[202] Gu Z., Zhu S., Yan L., Zhao F., Zhao Y. (2019). Graphene-Based Smart Platforms for Combined Cancer Therapy. Adv. Mater..

[203] Du Z., Zhang X., Guo Z., Xie J., Dong X., Zhu S., Du J., Gu Z., Zhao Y. (2018). X-Ray-Controlled Generation of Peroxynitrite Based on Nanosized LiLuF(4): Ce(3+) Scintillators and their Applications for Radiosensitization. Adv. Mater..

[204] Chen H., Gu Z., An H., Chen C., Chen J., Cui R., Chen S., Chen W., Chen X., Chen X. (2018). Precise nanomedicine for intelligent therapy of cancer. Sci. China Chem..

[205] Bobo D., Robinson K.J., Islam J., Thurecht K.J., Corrie S.R. (2016). Nanoparticle-based medicines: A review of FDA-approved materials and clinical trials to date. Pharm. Res..

[206] Lim Z.-Z.J., Li J.-E.J., Ng C.-T., Yung L.-Y.L., Bay B.-H. (2011). Gold nanoparticles in cancer therapy. Acta Pharmacol. Sin..

[207] Muddineti O.S., Ghosh B., Biswas S. (2015). Current trends in using polymer coated gold nanoparticles for cancer therapy. Int. J. Pharm..

[208] Tabassam Q., Mehmood T., Raza A.R., Ullah A., Saeed F., Anjum F.M. (2020). Synthesis, Characterization and Anti-Cancer Therapeutic Potential of Withanolide-A with 20nm sAuNPs Conjugates Against SKBR3 Breast Cancer Cell Line. Int. J. Nanomed..

[209] Wang D., Naydenov N.G., Dozmorov M.G., Koblinski J.E., Ivanov A.I. (2020). Anillin regulates breast cancer cell migration, growth, and metastasis by non-canonical mechanisms involving control of cell stemness and differentiation. Breast Cancer Res..

[210] Devanand Venkatasubbu G., Ramasamy S., Ramakrishnan V., Kumar J. (2013). Folate targeted PEGylated titanium dioxide nanoparticles as a nanocarrier for targeted paclitaxel drug delivery. Adv. Powder Technol..

[211] Yamaguchi S., Kobayashi H., Narita T., Kanehira K., Sonezaki S., Kudo N., Kubota Y., Terasaka S., Houkin K. (2011). Sonodynamic therapy using water-dispersed TiO_2_-polyethylene glycol compound on glioma cells: Comparison of cytotoxic mechanism with photodynamic therapy. Ultrason. Sonochem..

[212] Wang R., Hashimoto K., Fujishima A., Chikuni M., Kojima E., Kitamura A., Shimohigoshi M., Watanabe T. (1997). Light-induced amphiphilic surfaces. Nature.

[213] Yin Z.F., Wu L., Yang H.G., Su Y.H. (2013). Recent progress in biomedical applications of titanium dioxide. Phys. Chem. Chem. Phys..

[214] Xu P., Wang R., Ouyang J., Chen B. (2015). A new strategy for TiO_2_ whiskers mediated multi-mode cancer treatment. Nanoscale Res. Lett..

[215] Liu E., Zhou Y., Liu Z., Li J., Zhang D., Chen J., Cai Z. (2015). Cisplatin loaded hyaluronic acid modified TiO 2 nanoparticles for neoadjuvant chemotherapy of ovarian cancer. J. Nanomater..

[216] Kulshrestha S., Qayyum S., Khan A.U. (2017). Antibiofilm efficacy of green synthesized graphene oxide-silver nanocomposite using Lagerstroemia speciosa floral extract: A comparative study on inhibition of gram-positive and gram-negative biofilms. Microb. Pathog..

[217] Rajkumari J., Magdalane C.M., Siddhardha B., Madhavan J., Ramalingam G., Al-Dhabi N.A., Arasu M.V., Ghilan A.K.M., Duraipandiayan V., Kaviyarasu K. (2019). Synthesis of titanium oxide nanoparticles using Aloe barbadensis mill and evaluation of its antibiofilm potential against Pseudomonas aeruginosa PAO1. J. Photochem. Photobiol. B.

[218] Madadi Z., Bagheri Lotfabad T. (2016). Aqueous Extract of Acanthophyllum laxiusculum Roots as a Renewable Resource for Green synthesis of nano-sized titanium dioxide using Sol-gel Method. Adv. Ceram. Prog..

[219] Kashale A.A., Gattu K.P., Ghule K., Ingole V.H., Dhanayat S., Sharma R., Chang J.-Y., Ghule A.V. (2016). Biomediated green synthesis of TiO_2_ nanoparticles for lithium ion battery application. Compos. B Eng..

[220] Bischoff B.L., Anderson M.A. (1995). Peptization Process in the Sol-Gel Preparation of Porous Anatase (TiO_2_). Chem. Mater..

[221] Reinke M., Ponomarev E., Kuzminykh Y., Hoffmann P. (2015). Combinatorial Characterization of TiO_2_ Chemical Vapor Deposition Utilizing Titanium Isopropoxide. ACS Comb. Sci..

[222] Sakai H., Kawahara H., Shimazaki M., Abe M. (1998). Preparation of Ultrafine Titanium Dioxide Particles Using Hydrolysis and Condensation Reactions in the Inner Aqueous Phase of Reversed Micelles:  Effect of Alcohol Addition. Langmuir.

[223] Chen H., Wang Y., Dong S. (2007). An Effective Hydrothermal Route for the Synthesis of Multiple PDDA-Protected Noble-Metal Nanostructures. Inorg. Chem..

[224] Leshuk T., Parviz R., Everett P., Krishnakumar H., Varin R.A., Gu F. (2013). Photocatalytic activity of hydrogenated TiO_2_. ACS Appl. Mater. Interfaces.

[225] Schneider J., Matsuoka M., Takeuchi M., Zhang J., Horiuchi Y., Anpo M., Bahnemann D.W. (2014). Understanding TiO_2_ photocatalysis: Mechanisms and materials. Chem. Rev..

[226] Anderson C., Bard A.J. (1997). Improved Photocatalytic Activity and Characterization of Mixed TiO_2_/SiO_2_ and TiO_2_/Al_2_O_3_ Materials. J. Phys. Chem..

[227] Benavides J.A., Trudeau C.P., Gerlein L.F., Cloutier S.G. (2018). Laser Selective Photoactivation of Amorphous TiO_2_ Films to Anatase and/or Rutile Crystalline Phases. ACS Appl. Energy Mater..

[228] Carta D., Salaoru I., Khiat A., Regoutz A., Mitterbauer C., Harrison N.M., Prodromakis T. (2016). Investigation of the Switching Mechanism in TiO_2_-Based RRAM: A Two-Dimensional EDX Approach. ACS Appl. Mater. Interfaces.

[229] Bai Y., Mora-Seró I., De Angelis F., Bisquert J., Wang P. (2014). Titanium Dioxide Nanomaterials for Photovoltaic Applications. Chem. Rev..

[230] Maheswari P., Harish S., Navaneethan M., Muthamizhchelvan C., Ponnusamy S., Hayakawa Y. (2020). Bio-modified TiO(2) nanoparticles with *Withania somnifera*, Eclipta prostrata and Glycyrrhiza glabra for anticancer and antibacterial applications. Mater. Sci. Eng. C Mater. Biol. Appl..

[231] Al-Shabib N.A., Husain F.M., Qais F.A., Ahmad N., Khan A., Alyousef A.A., Arshad M., Noor S., Khan J.M., Alam P. (2020). Phyto-Mediated Synthesis of Porous Titanium Dioxide Nanoparticles From *Withania somnifera* Root Extract: Broad-Spectrum Attenuation of Biofilm and Cytotoxic Properties Against HepG2 Cell Lines. Front. Microbiol..

[232] Xu L., Wang Y.-Y., Huang J., Chen C.-Y., Wang Z.-X., Xie H. (2020). Silver nanoparticles: Synthesis, medical applications and biosafety. Theranostics.

[233] Abbasi E., Milani M., Fekri Aval S., Kouhi M., Akbarzadeh A., Tayefi Nasrabadi H., Nikasa P., Joo S.W., Hanifehpour Y., Nejati-Koshki K. (2016). Silver nanoparticles: Synthesis methods, bio-applications and properties. Crit. Rev. Microbiol..

[234] Mousavi S.M., Hashemi S.A., Ghasemi Y., Atapour A., Amani A.M., Savar Dashtaki A., Babapoor A., Arjmand O. (2018). Green synthesis of silver nanoparticles toward bio and medical applications: Review study. Artif. Cells Nanomed. Biotechnol..

[235] Kim D., Amatya R., Hwang S., Lee S., Min K.A., Shin M.C. (2021). BSA-Silver Nanoparticles: A Potential Multimodal Therapeutics for Conventional and Photothermal Treatment of Skin Cancer. Pharmaceutics.

[236] Tripathi D., Modi A., Narayan G., Rai S.P. (2019). Green and cost effective synthesis of silver nanoparticles from endangered medicinal plant Withania coagulans and their potential biomedical properties. Mater. Sci. Eng. C Mater. Biol. Appl..

[237] Gaurav I., Singh T., Thakur A., Kumar G., Rathee P., Kumari P., Sweta K. (2020). Synthesis, In-Vitro and In-Silico Evaluation of Silver Nanoparticles with Root Extract of *Withania somnifera* for Antibacterial Activity via Binding of Penicillin-Binding Protein-4. Curr. Pharm. Biotechnol..

[238] Kapoor S., Sood H., Saxena S., Chaurasia O.P. (2022). Green synthesis of silver nanoparticles using Rhodiola imbricata and *Withania somnifera* root extract and their potential catalytic, antioxidant, cytotoxic and growth-promoting activities. Bioprocess Biosyst. Eng..

[239] Bisht G., Rayamajhi S. (2016). ZnO Nanoparticles: A Promising Anticancer Agent. Nanobiomedicine.

[240] Zhang Y., Nayak T.R., Hong H., Cai W. (2013). Biomedical applications of zinc oxide nanomaterials. Curr. Mol. Med..

[241] Kołodziejczak-Radzimska A., Jesionowski T. (2014). Zinc Oxide-From Synthesis to Application: A Review. Materials.

[242] Vasuki K., Manimekalai R. (2019). NIR light active ternary modified ZnO nanocomposites for combined cancer therapy. Heliyon.

[243] Kumar J., Mitra M.D., Hussain A., Kaul G. (2019). Exploration of immunomodulatory and protective effect of *Withania somnifera* on trace metal oxide (zinc oxide nanoparticles) induced toxicity in Balb/c mice. Mol. Biol. Rep..

[244] Ventola C.L. (2017). Progress in nanomedicine: Approved and investigational nanodrugs. Pharm. Ther..

[245] Ventola C.L. (2012). The nanomedicine revolution: Part 1: Emerging concepts. Pharm. Ther..

[246] Fenske D.B., Cullis P.R. (2008). Liposomal nanomedicines. Expert. Opin. Drug Deliv..

[247] Min Y., Caster J.M., Eblan M.J., Wang A.Z. (2015). Clinical translation of nanomedicine. Chem. Rev..

[248] Zhang C., Yan L., Wang X., Zhu S., Chen C., Gu Z., Zhao Y. (2020). Progress, challenges, and future of nanomedicine. Nano Today.

[249] Sercombe L., Veerati T., Moheimani F., Wu S.Y., Sood A.K., Hua S. (2015). Advances and challenges of liposome assisted drug delivery. Front. Pharmacol..

[250] Guo X., Szoka F. (2001). Steric stabilization of fusogenic liposomes by a low-pH sensitive PEG− diortho ester− lipid conjugate. Bioconjugate Chem..

[251] Bibi S., Lattmann E., Mohammed A.R., Perrie Y. (2012). Trigger release liposome systems: Local and remote controlled delivery?. J. Microencapsul..

[252] Woodle M.C., Lasic D.D. (1992). Sterically stabilized liposomes. Biochim. Biophys. Acta (BBA)-Rev. Biomembr..

[253] Allen T.M., Hansen C., Rutledge J. (1989). Liposomes with prolonged circulation times: Factors affecting uptake by reticuloendothelial and other tissues. Biochim. Biophys. Acta Biomembr..

[254] Hua S., Wu S.Y. (2013). The use of lipid-based nanocarriers for targeted pain therapies. Front. Pharmacol..

[255] Kraft J.C., Freeling J.P., Wang Z., Ho R.J. (2014). Emerging research and clinical development trends of liposome and lipid nanoparticle drug delivery systems. J. Pharm. Sci..

[256] Bulbake U., Doppalapudi S., Kommineni N., Khan W. (2017). Liposomal formulations in clinical use: An updated review. Pharmaceutics.

[257] Liu D., He C., Wang A.Z., Lin W. (2013). Application of liposomal technologies for delivery of platinum analogs in oncology. Int. J. Nanomed..

[258] Ko A.H., Tempero M.A., Shan Y.S., Su W.C., Lin Y.L., Dito E., Ong A., Wang Y.W., Yeh C.G., Chen L.T. (2013). A multinational phase 2 study of nanoliposomal irinotecan sucrosofate (PEP02, MM-398) for patients with gemcitabine-refractory metastatic pancreatic cancer. Br. J. Cancer..

[259] Koudelka Š., Turánek J. (2012). Liposomal paclitaxel formulations. J. Control. Release.

[260] Deeken J.F., Slack R., Weiss G.J., Ramanathan R.K., Pishvaian M.J., Hwang J., Lewandowski K., Subramaniam D., He A.R., Cotarla I. (2013). A phase I study of liposomal-encapsulated docetaxel (LE-DT) in patients with advanced solid tumor malignancies. Cancer Chemother. Pharmacol..

[261] Belfiore L., Saunders D.N., Ranson M., Thurecht K.J., Storm G., Vine K.L. (2018). Towards clinical translation of ligand-functionalized liposomes in targeted cancer therapy: Challenges and opportunities. J. Control. Release.

[262] Sapra P., Tyagi P., Allen T.M. (2005). Ligand-targeted liposomes for cancer treatment. Curr. Drug Deliv..

[263] Cheng C.-C., Chang F.-C., Kao W.-Y., Hwang S.-M., Liao L.-C., Chang Y.-J., Liang M.-C., Chen J.-K., Lee D.-J. (2016). Highly efficient drug delivery systems based on functional supramolecular polymers: In vitro evaluation. Acta Biomater..

[264] Hassan S., Prakash G., Ozturk A.B., Saghazadeh S., Sohail M.F., Seo J., Dokmeci M.R., Zhang Y.S., Khademhosseini A. (2017). Evolution and clinical translation of drug delivery nanomaterials. Nano Today.

